# Identification and validation of key biomarkers based on RNA methylation genes in sepsis

**DOI:** 10.3389/fimmu.2023.1231898

**Published:** 2023-08-28

**Authors:** Qianqian Zhang, Xiaowei Bao, Mintian Cui, Chunxue Wang, Jinlu Ji, Jiongjie Jing, Xiaohui Zhou, Kun Chen, Lunxian Tang

**Affiliations:** ^1^ Department of Internal Emergency Medicine, Shanghai East Hospital, School of Medicine, Tongji University, Shanghai, China; ^2^ School of Medicine, Tongji University, Shanghai, China; ^3^ Translational Medical Center for Stem Cell Therapy, Institute for Regenerative Medicine, Shanghai East Hospital, School of Life Sciences and Technology, Tongji University, Shanghai, China; ^4^ Research Center for Translational Medicine, Shanghai Heart Failure Research Center, Shanghai East Hospital, Tongji University, Shanghai, China; ^5^ Shanghai Key Laboratory of Signaling and Disease Research, Frontier Science Center for Stem Cell Research, School of Life Sciences and Technology, Tongji University, Shanghai, China

**Keywords:** sepsis, RNA methylation, machine learning, unsupervised clustering, biomarkers

## Abstract

**Background:**

RNA methylation is closely involved in immune regulation, but its role in sepsis remains unknown. Here, we aim to investigate the role of RNA methylation-associated genes (RMGs) in classifying and diagnosing of sepsis.

**Methods:**

Five types of RMGs (m1A, m5C, m6Am, m7G and Ψ) were used to identify sepsis subgroups based on gene expression profile data obtained from the GEO database (GSE57065, GSE65682, and GSE95233). Unsupervised clustering analysis was used to identify distinct RNA modification subtypes. The CIBERSORT, WGCNA, GO and KEGG analysis were performed to explore immune infiltration pattern and biological function of each cluster. RF, SVM, XGB, and GLM algorithm were applied to identify the diagnostic RMGs in sepsis. Finally, the expression levels of the five key RMGs were verified by collecting PBMCs from septic patients using qRT-PCR, and their diagnostic efficacy for sepsis was verified in combination with clinical data using ROC analysis.

**Results:**

Sepsis was divided into three subtypes (cluster 1 to 3). Cluster 1 highly expressed *NSUN7* and *TRMT6*, with the characteristic of neutrophil activation and upregulation of MAPK signaling pathways. Cluster 2 highly expressed *NSUN3*, and was featured by the regulation of mRNA stability and amino acid metabolism. *NSUN5* and *NSUN6* were upregulated in cluster 3 which was involved in ribonucleoprotein complex biogenesis and carbohydrate metabolism pathways. In addition, we identified that five RMGs (*NSUN7*, *NOP2*, *PUS1*, *PUS3* and *FTO*) could function as biomarkers for clinic diagnose of sepsis. For validation, we determined that the relative expressions of *NSUN7*, *NOP2*, *PUS1* and *PUS3* were upregulated, while *FTO* was downregulated in septic patients. The area under the ROC curve (AUC) of *NSUN7*, *NOP2*, *PUS1*, *PUS3* and *FTO* was 0.828, 0.707, 0.846, 0.834 and 0.976, respectively.

**Conclusions:**

Our study uncovered that dysregulation of RNA methylation genes (m1A, m5C, m6Am, m7G and Ψ) was closely involved in the pathogenesis of sepsis, providing new insights into the classification of sepsis endotypes. We also revealed that five hub RMGs could function as novel diagnostic biomarkers and potential targets for treatment.

## 1 Introduction

Sepsis is defined as life-threatening organ dysfunction caused by a dysregulated host response to infection (1). Although huge advance has been made in the treatment of sepsis, the mortality rate remains high, amounting to about 30% to 50% (2). According to the Surviving Sepsis Campaign International Guidelines, antibiotic treatment should be initiated within one hour after sepsis onset, delay in antibiotic therapy is closely associated with mortality (3). However, early recognition of sepsis remains a big clinical challenge due to its heterogeneity and complexity in terms of manifestations and populations (4, 5). Therefore, developing new biomarkers and classifying sepsis are critical for its early diagnosis and treatment.

The identification and classification of sepsis was previously based on clinical features or biomarkers. An increasing number of biomarkers for sepsis have been revealed in different independent studies including inflammatory cytokines, chemokines, complement system, metabolic genes, damage associated molecular patterns (DAMPs), non-coding RNAs, cell membrane receptors and proteins which facilitate sepsis diagnosis, and allow an early intervention (6, 7). However, due to the individual differences and pathophysiological complexity of sepsis, using a single biomarker in clinical settings does not achieve efficient diagnosis. Therefore, exploring a panel of specific and sensitive biomarkers or models combining biomarkers and clinical data to augment diagnosis and stratify sepsis patients is an urgent need.

RNA methylation is a reversible chemical modification by adding or removing methyl group on adenosine (A) or cytosine(C). It is the most abundant RNA modification in eukaryotes and prokaryotes (8, 9). More than 100 types of RNA methylation modifications have been identified in messenger RNA (mRNA) and noncoding RNA (ncRNA), including N6-methyladenosine (m6A), N1-methyladenosine (m1A), 5-methylcytidine (m5C), N7-methylguanosine (m7G), 2’-O-dimethyladenosine (m6Am), Pseudouridine (Ψ) and so on (9–11). This dynamic process is regulated by various enzymes and binding proteins including methyltransferases, demethylases and modified RNA binding proteins which are known as “writers”, “erasers” and “readers”, respectively. RNA methylation was revealed to be closely associated with clustering of various diseases (12) and acts as the potential biomarker for diagnosis or treatment in cancers, autoimmune diseases, cardiovascular diseases, and viral infections (13–16). Recently, sepsis was classified into three different subtypes with different prognostic outcomes according to the expression of m6A RNA methylation regulatory genes (17). Methyltransferase-like 3 (*METTL3*), one of the key m6A RNA methylation writers, could work as a potential therapeutic target for LPS-induced endotoxemia (18).

Peripheral blood mononuclear cells (PBMCs), including lymphocytes, macrophages, dendritic cell, monocytes and so on (19), play a crucial role in sepsis. Pathogens can rapidly induce inflammatory response by activating PBMCs to produce substantial pro-inflammatory mediators, while excessive inflammatory response in turn disrupts the function of PBMCs (20). Meanwhile, the upregulation of anti-inflammatory mediators and immunosuppressive factors can induce apoptosis and pyroptosis of PBMCs (21). Epigenetic modifications, particularly RNA methylations are important for regulating the function of immune cells in sepsis. *ALKBH5*, a m6A erase, was significantly downregulated in sepsis, and *ALKBH5* deficiency would suppress the expression of the chemokine receptor *CXCR2*, which inhibited neutrophil migration and inflammation during bacterial infection (22). Overexpression of *YTHDF1* in macrophages could upregulate WW domain containing E3 Ubiquitin protein ligase 1 (*WWP1*) and thereby alleviate sepsis through promoting *NLRP3* ubiquitination and inhibiting caspase-1-dependent pyroptosis (23).However, the systemic investigation of the role of RNA methylation including m1A/m6Am/m5C/m7G/Ψ in sepsis is lacking. The important role of RNA methylation in pathogenesis of various diseases indicates the possibility to evaluate whether RNA methylation (m1A, m5C, m6Am, m7G and Ψ) associated genes could work as biomarkers for sepsis classification and early diagnosis.

In this study, by integrated analysis of the expression of RNA methylation-associated genes (RMGs) from public datasets, we divided sepsis into three subclusters, and each cluster displayed distinct immune cells infiltration and physiological functions. In addition, through machine learning, we identified five hub RMGs that were closely related to the diagnosis of sepsis. Finally, we validated the mRNA levels of five hub RMGs in PBMCs of septic patients and assessed their diagnostic value for sepsis in clinical settings. These findings elucidate the important role of RNA methylation in the diagnosis of sepsis and provide a theoretical and clinical foundation for further investigation into the role of RMGs in sepsis.

## 2 Methods

### 2.1 Data sources and study selection

**Figure 1:**
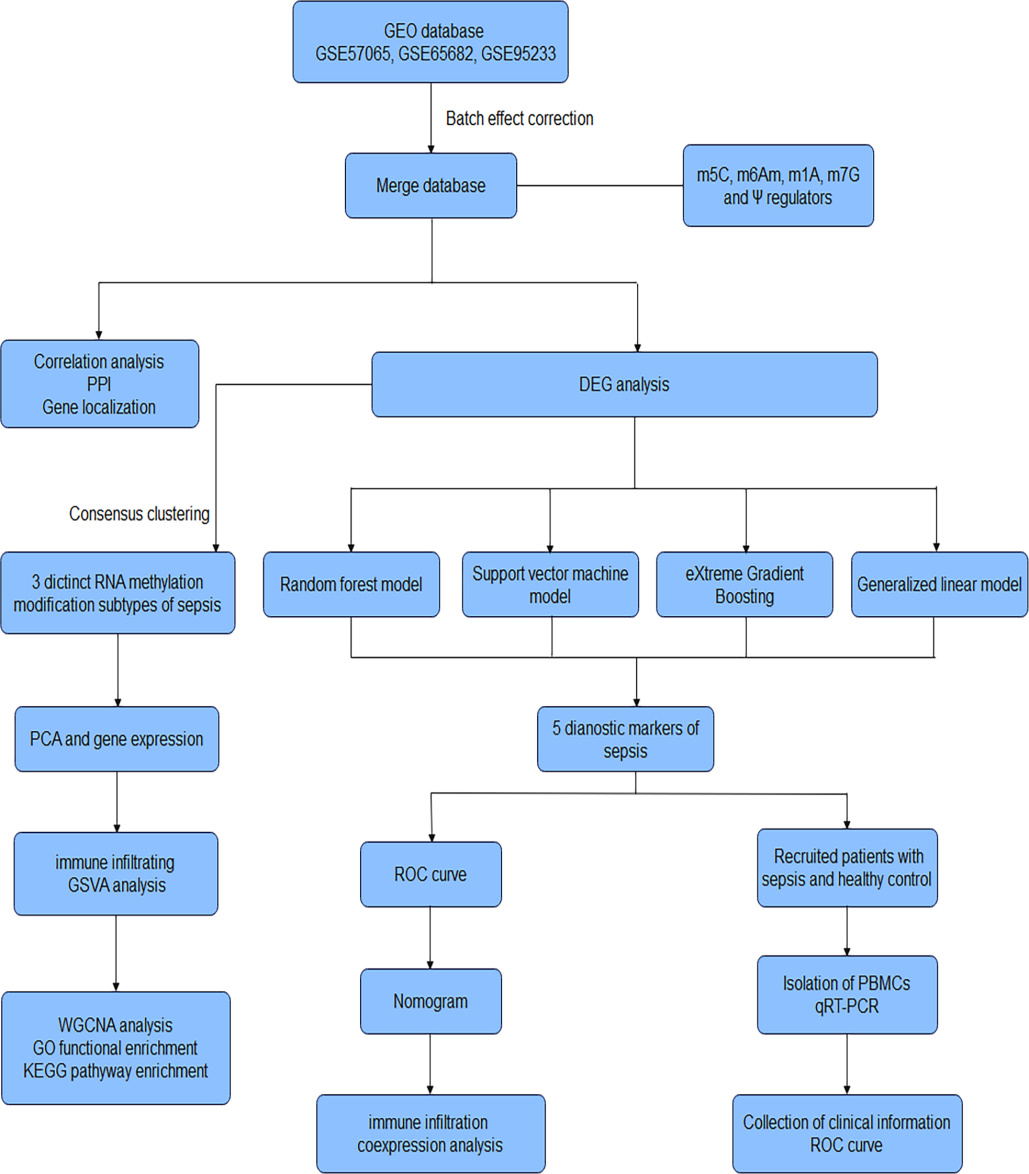
Workflow of the research. GEO Gene Expression Omnibus, m5C 5-methylcytosine, m6Am 2’-O-dimethyladenosine, m1A N1-methyladenosine, m7G N7-methylguanosine, Ψ Pseudouridine, PPI protein-protein interaction, DEG Differentially expressed gene, PCA Principal Component Analysis, GSVA Gene Set Variation Analysis, WGCNA Weighted gene co-expression network analysis, GO Gene Ontology, KEGG Kyoto Encyclopedia of Genes and Genomes, ROC receiver operating characteristic curve, qRT-PCR Quantitative reverse-transcription polymerase chain reaction, PBMCs peripheral blood mononuclear cells.

The research strategy is presented in **Figure 1**. We conducted a search of the GEO database (http://www.ncbi.nlm.nih.gov/geo/) (24) for expression microarrays related to sepsis. We included datasets from clinical studies of sepsis in adults using peripheral blood samples. Retrieved gene expression profile data for sepsis patients (GSE57065, GSE65682 and GSE95233) using the R package GEOquery, merged the gene expression groups, and split them into sepsis and healthy control groups. Details of the datasets are shown in **Table S1**.

### 2.2 Collecting RMGs *via* systematic review

We first compiled a list of 40 RNA methylation regulators in 5 categories (including m5C, m6Am, m1A, m7G and Ψ) from published research (25–27), which included writers, readers, and erasers, showed in **Table S2**. We then obtained a total of 34 RMGs by screening gene expression with detectable expression in the GEO datasets.

### 2.3 Acquisition of data and differentially expressed genes

All datasets were downloaded and outputs from mRNA array were normal-exponential background corrected and quantile normalized between arrays using the limma R package (28). Expression was normalized using a weighted linear regression, and precision weights were multiplied with corresponding log2 values to yield final gene expression values. Genes with an absolute log fold change (*log_2_FC*) > 1 and adjusted *p*-value < 0.05 were considered up-regulated, and those with *log_2_FC* < - 1 and adjusted *p*-value < 0.05 were considered down-regulated. We also performed Spearman correlation analysis to investigate the relationship between each gene. Functional interactions of the RMGs were explored using the Search Tool for Retrieval of Interacting Genes (STRING 11.0, http://string-db.org/cgi/input.pl), and a Protein-protein interaction (PPI) network was constructed. We visualized the chromosomal localization of RMGs using the R circos package (29).

### 2.4 Exploration of RMGs induced molecular subtypes of sepsis

The consensus clustering algorithm works by performing clustering on the same dataset multiple times, using various clustering methods or different parameter settings. The next step is to create a consensus matrix or a consensus function that measures the degree of agreement among the individual clustering solutions. This consensus matrix represents the overall consensus for each sample across different clustering methods, and is used to identify robust and stable clusters. We used consensus k means clustering to identify highly heterogeneous RNA methylation subtypes in sepsis based on RNA methylation regulator expression profiles. Clustering was performed using 100 iterations, with each iteration containing 80% of samples. We determined the optimal number of clusters using CDF curves of the consensus score, clear separation of the consensus matrix heatmaps, characteristics of the consensus cumulative distribution function plots, and adequate pairwise-consensus values between cluster members. To assess the expression distribution of RNA methylation regulators in different subtypes, we utilized moderated t-tests.

### 2.5 Analysis of immune infiltrating cells among subtypes in sepsis

To better understand the situation of infiltrating immune cells in C1-C3 subtypes, we used the CIBERSORT algorithm. CIBERSORT is a bioinformatics tool used to deconvolute relative cell type proportions and gene expression profiles from bulk RNA sequencing datasets. The Gene Set Variation Analysis (GSVA) R software package was downloaded from http://www.bioconductor.org, and the pathway with differential enrichment in the three groups was obtained. GSVA is used to evaluate the activity or enrichment of biological pathways, gene sets, or functional signatures in individual samples from high-throughput gene expression data, such as RNA-Seq. The analysis quantifies the degree to which a specific gene set is upregulated or downregulated in each individual sample, generating a continuous enrichment score for each sample-gene set pair.

### 2.6 Assessing the heterogeneity of biological function among subtypes

To find gene-sets significantly correlated to RNA methylation subtypes, we used weighted correlation network analysis (WGCNA) with the WGCNA R package (30). WGCNA is a powerful bioinformatics method used to analyze gene expression data and identify co-expression patterns among genes. Genes from modules highly associated with RNA methylation subtypes (the maximum correlation coefficient and *P* < 0.05) were selected for further Gene ontology (GO) and Kyoto Encyclopedia of Genes and Genomes (KEGG) analysis, to investigate the biological functions and signaling pathways involved in sepsis.

### 2.7 Construction and assessment of RF, GLM, SVM and XGB model

We created Random forest model (RF), support vector machine model (SVM), eXtreme Gradient Boosting (XGB) and generalized linear model (GLM) using the integrated dataset based on RMGs. The RF, GLM, SVM and XGB models all belong to machine learning model which is used to assess the importance of variables. Here, we applied them in screening of diagnostic genes for sepsis. The different classes of sepsis were utilized as the response variable, and the RMGs were used as explanatory variables. We then used the explain feature of the “DALEX” package in R to analyze the aforementioned four models and residual distribution, and the receiver operating characteristics (ROC) was plotted to select the best model using the integrated dataset. Finally, to evaluate the diagnostic efficacy of the five most important explanatory variables in the best model, we calculated ROC curve using the ‘survivalROC’ package in the three databases respectively. The AUC was used to evaluate the diagnostic genes: 0.5-0.7 (moderate), 0.7-0.8 (good), and >0.9 (excellent).

### 2.8 Construction and validation of a nomogram model for sepsis diagnosis

Nomogram model is a visual aid that combines multiple variables and assigns them a weighted score to estimate the likelihood of a specific outcome. Using the “rms” package, we established a nomogram model to predict the occurrence of sepsis. “Points” indicate the score of the corresponding factor below, and “Total Points” indicate the summation of all the scores of factors above. We then used calibration curves to assess the predictive power of the nomogram model. Finally, we evaluated the clinical value of the model using decision curve analysis.

### 2.9 Construction of hub RMGs co-expression and miRNA network

The co-expressed gene network of hub RMGs was constructed using COEXPEDIA (https://www.coexpedia.org). The potential miRNAs targeting key RMGs were downloaded from miRanda (https://www.cs.kent.ac.uk/people/staff/dat/miranda/) and miRDB (http://mirdb.org/) databases, and overlapping miRNAs were selected to depict network. The final gene-miRNA and the co-expression network associated with sepsis were visualized using Cytoscape software.

### 2.10 Septic patients and controls

This study included 39 adult patients from our hospital, consisting of 26 septic patients diagnosed with sepsis according to the Third International Consensus Definitions for Sepsis and Septic Shock (Sepsis-3) (1), and 13 healthy controls. Healthy volunteers were those who came to East Hospital for routine physical examination. Peripheral blood samples and corresponding clinical data were collected upon admission. The characteristics of the patients are shown in **Table 1**. PBMCs were isolated within 4 hours after collection.

**Table 1:** Baseline characteristic of septic patients and healthy volunteers.

Baseline Characteristics	Septic Shock (n=11)	Sepsis (n=15)	Healthy Volunteers (n=13)	P value in the first two groups	P value in three groups
	Mean ± SD/Median (IQR)				
Age (years)	79.45 ± 12.09	70.47 ± 13.75	66.46 ± 3.431	0.0965	0.0501
Sex[male (%)]	7 (63.64)	13 (86.67)	6 (46.15)	0.0740	0.0772
Comorbidities[n] (%)					
Chronic Pulmonary Disease	2 (5.41)	1 (3.57)	–	NS.	–
Chronic Kidney Disease	6 (16.22)	4 (14.29)	–	NS.	–
Cardiovascular Disease	8 (21.62)	4 (14.29)	–	NS.	–
Hepatopathy	6 (16.22)	5 (17.86)	–	NS.	–
Diabetes	2 (5.41)	7 (25.00)	–	NS.	–
Hypertension	8 (21.62)	6 (21.43)	–	NS.	–
Hyperlipidemia	1 (2.70)	0 (0.00)	–	NS.	–
Coagulation Dysfunction	3 (8.11)	1 (3.57)	–	NS.	–
Malignant Tomor	1 (2.70)	0 (0.00)	–	NS.	–
Focus of infection[n] (%)					
Gastrointestinal	6 (54.55)	8 (50.00)	–	NS.	–
Pulmonary	4 (36.36)	4 (25.00)	–	NS.	–
Urinary tract	1 (9.09)	3 (18.75)	–	NS.	–
Skin or soft tissue	0 (0.00)	1 (6.25)	–	NS.	–
APACHE II score	23.44 ± 7.70	10.45 ± 3.80	–	0.0001	–
SOFA score on day 1	10.45 ± 3.80	5.66 ± 3.22	–	0.0020	–
Length of ICU stay (days)	8.46 (0.00-19.00)	6.87 (0.00-31.00)	–	0.6113	–
Length of Hospital stay (days)	13.00 (1.00-30.00)	19.60 (6.00-42.00)	–	0.1362	–
Mechanical ventilation percentage	10 (90.90)	3 (20.00)	–	0.0010	–
Mechanical ventilation time (days)	6.64 (0.00-19.00)	1.33 (0.00-13.00)	–	0.0177	–
7-days mortality	6 (54.55)	0 (0.00)	–	0.0020	–
14-days mortality	7 (63.64%)	1 (6.67%)	–	0.0038	–
28-days mortality	8 (72.73%)	1 (6.67%)	–	0.0008	–

ICU, intensive care unit;. IQR, inter quartile range; APACHE II, score acute physiology and chronic health evaluation II score; SOFA, sequential organ failure assessment P values were calculated by Mann-Whitney U test, Students’ t-test or one-way analysis of variance (one-way ANOVA), and c² test or Fisher’s exact test, as appropriate. P values below 0.05 indicates statistical significance. NS, no significance.

This study was approved by the Research Ethics Board of East Hospital, Tongji University (Shanghai, China). Written informed consent was obtained from all recruited patients or their authorized family members.

### 2.11 Isolation of PBMCs

Blood samples in EDTA-containing tubes were centrifuged at 3000 rpm for 5 min at room temperature. Serum was removed, and PBMCs were separated from the remaining blood using Ficoll-Paque density gradient centrifugation, following the instruction manual (Solarbio, Cat. NO. P8610). The PBMCs were then stored at -80°C until further testing.

### 2.12 Real-time quantitative PCR

RNA was extracted from cells using TRIzol (Gene Copoeia, MD, USA), and 1 µg total RNA from each sample was reverse transcribed to cDNA using PrimeScript^®^ RT Master Mix (Takara, Cat. NO. RR036A). SYBR^®^ Green (Applied Biosystems™, Cat. NO. 4309155) was used for qRT-PCR analysis. The ΔCt method was used to analyze mRNA levels relative to glyceraldehyde-3-phosphate dehydrogenase (GAPDH). The primer sequences were listed in **Table S3**.

### 2.13 Statistical analysis

GraphPad Prism 9.4 (GraphPad Inc, San Diego, USA) was used for statistical analyses. Normally distributed data were compared using Student’s t-test or one-way analysis of variance (one-way ANOVA), and the results were shown as the mean ± SD. Nonnormally distributed data were analyzed using the non-parametric Mann-Whitney U test, and the results were expressed as the median and interquartile range (IQR). Categorical variables were compared with the chi-square or Fisher’s exact test, and the results were shown as numbers and percentages. All statistical tests were two-sided. *P* < 0.05 was regarded as statistically significant.

## 3 Results

### 3.1 Overall expression patterns of RNA methylation genes in PBMCs from septic patients

**Figure 2:**
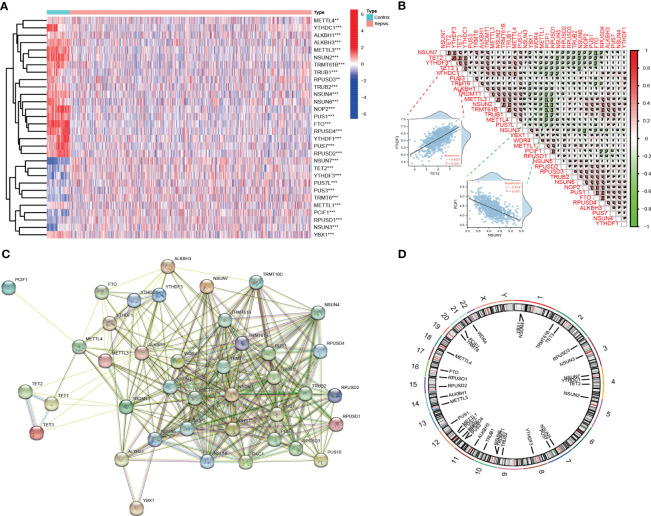
Landscape of expression and genetic variation of RNA methylation regulators in sepsis. **(A)** The heatmap shows the expression of RMGs obtained from integrated gene expression matrix. In the heatmap, rows represent transcripts, and columns represent samples (Medium Turquoise represents normal profiles, LightPink represents disease profiles). Red represents significantly upregulated genes and blue represents significantly downregulated genes in the samples. **P < 0.01, and ***P < 0.001 *vs*. The healthy group. **(B)** Spearman correlation analysis of the studied RMGs, the two scatter plots displayed the most positively or negatively correlated RMGs. **(C)** The protein-protein interaction between RMGs. **(D)** The chromosomal locations of RMGs across 23 chromosomes.

The overall strategy of this study was presented in **Figure 1**. To analyze the expression of RMGs in PBMCs from patients with sepsis, we first performed differential expressed genes (DEGs) analysis of the integrated gene expression matrix using the limma package. DEGs in PBMCs from healthy donors and septic patients were shown in heatmap (**Figure 2A**). Most of the genes that mediate one certain type of RNA methylation modifications displayed a similar expression pattern. For instance, genes mediating RNA Ψ modification (*TRUB1*, *TRUB2*, *PUS1*, *RPUSD3*, *RPUSD4*, *PUS7*, *RPUSD2*) were mainly suppressed in sepsis (**Figure 2A**, **Table S4**).

We then investigated the correlation among RMGs by using Spearman’s correlation analysis and found that there was a significantly close correlation among these differentially expressed RMGs. Of note, *YTHDF3* expression was positively correlated with the expression of *TET2*, whereas *PCIF1* exhibited a negative correlation with most other regulators (**Figure 2B**). The correlated expression among RMGs suggested their functional synergy or antagonism in the pathogenesis of sepsis. Moreover, we performed a PPI network analysis of RMGs. Proteins with an interaction score ≥0.4 were selected and visualized (**Figure 2C**). The network comprised 40 nodes and 257 edges, representing genes and interactions between genes, respectively (**Table S5**), also indicating the functional association in sepsis. The chromosomal locations of RMGs were depicted in **Figure 2D**.

### 3.2 Identification of three subtypes of sepsis based on RMGs expression characteristics

**Figure 3:**
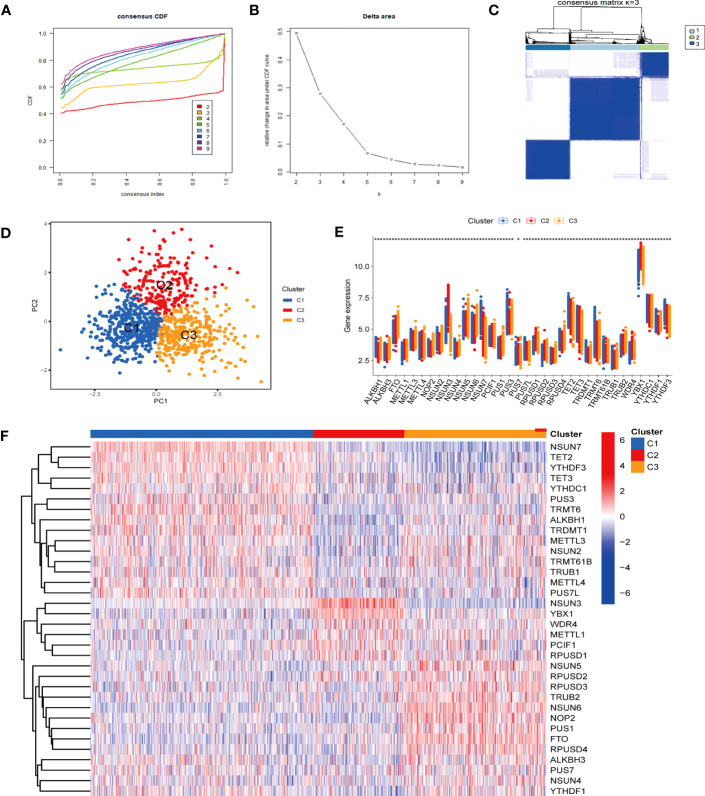
Identification of optimal sepsis subtypes based on the expression of RMGs. **(A)** The CDF curves based on different subtype numbers (k=2, 3, 4, 5, 6, 7, 8, 9) are represented, and each curve is associated with a unique color. CDF, cumulative distribution function curves. **(B)** The CDF Delta area curve of all samples. **(C)** Consensus heatmaps show a relativeiy stable partitioning of the samples at k = 3. **(D)** PCA is performed on different groups, where blue represents cluster 1, red represents cluster 2, and yellow represents cluster 3. PCA, principal component analysis. **(E)** The expression of RMGs among subtypes. *P < 0.05, **P < 0.01, ***P < 0.001. **(F)** The heatmap of the expression of RMGs between the C1, C2 and C3 groups.

Given that the differential expression levels of RNA modification genes and clustering stability, we employed consensus clustering, an unsupervised clustering method to obtain a robust ranking for subsequent analysis, and classified sepsis into three subtypes (cluster 1 to 3). The cumulative distribution function (CDF) plot displayed the consensus distributions for each cluster (**Figure 3A**). The delta area plot showed the relative change in the area under the CDF curve (**Figure 3B**). The CDF distribution was smoother when k = 3 or 4, while the increase in area under the CDF curve was relatively less at k = 4 than that at k = 3, suggesting that k = 3 was the optimal number of clusters. As shown in the Consensus matrix heatmap (**Figure 3C**, **S1**), 3 clusters showed clear boundaries, indicating good cluster stability over repeated iterations. The principal component analysis (PCA) results also indicated the justification of the three clusters (**Figure 3D**).

In addition, the expression of RMGs in each subtype displayed distinct patterns (*P* < 0.05) (**Figures 3E, F**). In cluster 1, *FTO* was relatively downregulated, and of all the highly expressed genes, *NSUN7* and *TRMT6* were most upregulated. These genes were involved in the regulation of m6Am, m5C and m1A RNA methylation modification. *NSUN3*, encoding a key methyltransferase for RNA m5C modification showed a significantly increased expression in cluster 2. In cluster 3, among the relatively highly expressed regulators, *NSUN5* and *NSUN6* significantly increased, which were also correlated with m5C modification. Those results indicated that m5C RNA methylation may play a role in sepsis classification.

Besides, we analyzed the basic clinical characteristics of sepsis patients in GSE65682 in 3 clusters (**Table S6**) and found significant differences in Molecular Diagnosis and Risk Stratification of Sepsis (MARS) endotypes among the three clusters (*P* < 0.001). In cluster 1, MARS 2 endotypes accounted for the highest proportion (42.44%). The proportion of MARS 1 (47.12%) was significantly higher in cluster 2 than that in the other two groups (*P* < 0.05). Cluster 3 was dominated by MARS 3 (35.56%). Previous studies have revealed that MARS 1 was consistently correlated to a poor outcome (31). But we did not observe significant differences in mortality among the three clusters (*P* = 0.074) (**Table S6**).

### 3.3 Three clusters differed in immune cell landscape and molecular pathway

**Figure 4:**
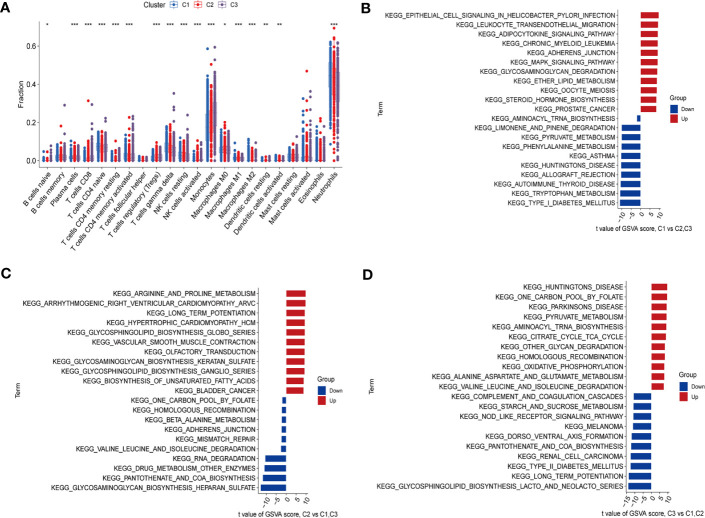
Immune cell infiltration and biological characteristics of three clusters. **(A)** Differences in infiltrating immune cells between the C1, C2 and C3 clusters. The upper and lower ends of the boxes indicate the interquartile range of values, with the lines in the boxes representing the median value and the dots indicating outliers. *P < 0.05; **P < 0.01; ***P < 0.001. **(B–D)** GSVA analysis conducted on different subtypes of sepsis. **(B)** Representative barplot showing the 11 relatively up-regulated and 10 down-regulated signaling pathways in C1 compared to C2 and C3. **(C)** Representative barplot showing the 11 relatively up-regulated and 10 down-regulated signaling pathways in C2 compared to C1 and C3. **(D)** Representative barplot showing the 11 relatively up-regulated and 10 down-regulated signaling pathways in C3 compared to C1 and C2.

By using CIBERSORT and GSVA analyses, we further identified a significant heterogeneity in terms of immune cell infiltration and molecular pathways among the three subtypes. The abundance and proportions of infiltrating immune cells differed among the three types, of which neutrophils and monocytes accounted for higher proportions as compared to the other immune cells (**Figure 4A**). In cluster 1, the frequencies of neutrophils and gamma delta T (γδT) cells were relatively increased compared to other clusters, while CD8 T cells, regulatory T cells (Tregs), activated NK cells were down-regulated (*P* < 0.05). The distribution of immune cells in cluster 2 and 3 showed relatively opposed patterns as compared to that in cluster 1 (*P* < 0.05). In cluster 3, CD8 T cells and monocytes were relatively upregulated, while γδT cells and neutrophils were down-regulated (*P* < 0.05). Immune cells that were up- or down-regulated in cluster 3 showed the same trend in cluster 2 with a less degree than those in cluster 3. Collectively, these results suggested that the three clusters have different immune infiltration in septic patients.

We then evaluated the different signaling pathways in these clusters. **Figures 4B–D** showed relatively up-regulated (11) and down-regulated (10) signaling pathways in the three sepsis subgroups. Compared to other subtypes, cluster 1 showed enhanced inflammatory signaling pathways activation, such as the Leukocyte transendothelial migration and MAPK signaling pathway. The Down-regulated pathways in cluster 1 were involved in amino acid metabolism (**Figure 4B**). In cluster 2, we found that up-regulated signaling pathways were associated with amino acids, glycosphingolipids and unsaturated fatty acids metabolism. Down-regulated signaling pathways were mainly related to maintaining the stability of the genome and amino acid metabolism (**Figure 4C**). In cluster 3, up-regulated signaling pathways were mainly involved in carbohydrate metabolism pathways. Down-regulated signaling pathways were related to inflammatory diseases (**Figure 4D**). These functional analyses indicated that septic patients with different RMG expression patterns have distinct molecular pathways.

### 3.4 Identification of key modules in three sepsis clusters by weighted gene co-expression network analysis

**Figure 5:**
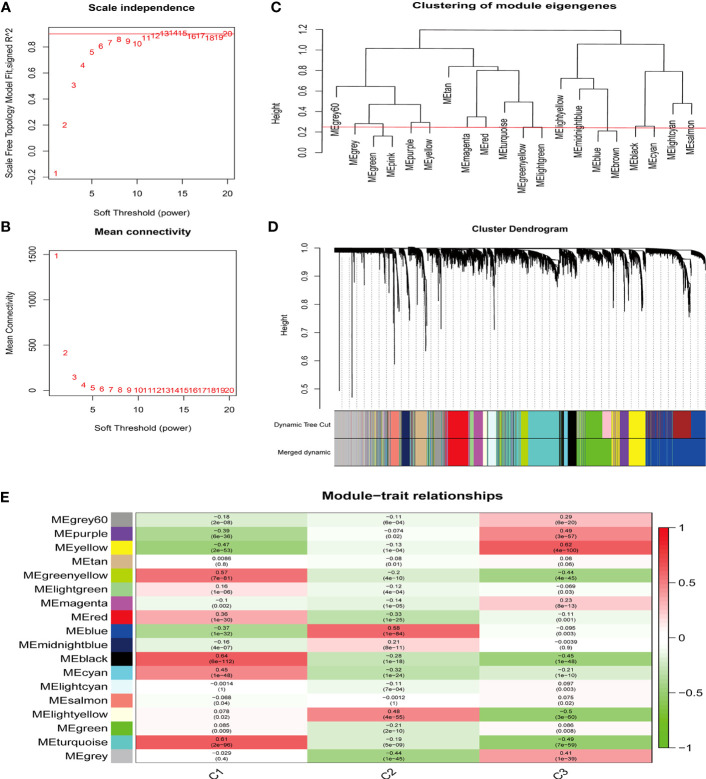
Identification of the key module by WGCNA. **(A, B)** The analysis of network topology for various soft thresholding powers of WGCNA. **(C)** Clustering dendrogram of module eigengenes. The red line indicates the cut height (0.25). **(D)** Hierarchical clustering dendrograms of co-expressed genes in identified modules are shown. Both dynamic and merged modules were identified. **(E)** WGCNA in the three sepsis subtypes. The 18 modules were validated and are designated by the different colors. The heatmap displays the correlation between feature vectors of 18 modules and three subtypes. The correlation coefficient in each cell represented the correlation between the gene module and the clusters, which decreases in size from red to green. The corresponding *P*-value is also annotated.

Next, we performed WGCNA to analyze the gene co-expression networks and identify biologically meaningful modules that corresponded to designated phenotype-related genes. A scale free co-expression network was established with the soft threshold power as 12 (scale-free *R* ^2 =^ 0.90) (**Figures 5A, B**) and cut height as 0.25 (**Figure 5C**). The cluster dendrogram was displayed in **Figure 5D**. WGCNA identified 18 modules in the sepsis cohort (**Figure 5E**). Cluster 1 was positively correlated with MEblack module (containing 390 genes) (**Table S7**), with a correlation coefficient of 0.64 and *P* value of 6.00E-112 (**Figure S2A**). Cluster 2 was positively associated with MEblue module (containing 1510 genes) (**Table S7**), with a correlation coefficient of 0.58 and *P* value of 1.00E-84 (**Figure S2B**). Cluster 3 was significantly positively correlated to MEyellow (containing 547 genes) (**Table S7**), with a correlation coefficient of 0.62 and *P* value of 4.00E-100 (**Figure S2C**). To identify genes highly correlated with the key module in each cluster, we set module membership (MM) > 0.8 and gene significance (GS) > 0.3, and results were shown in **Table S8**.

**Figure 6:**
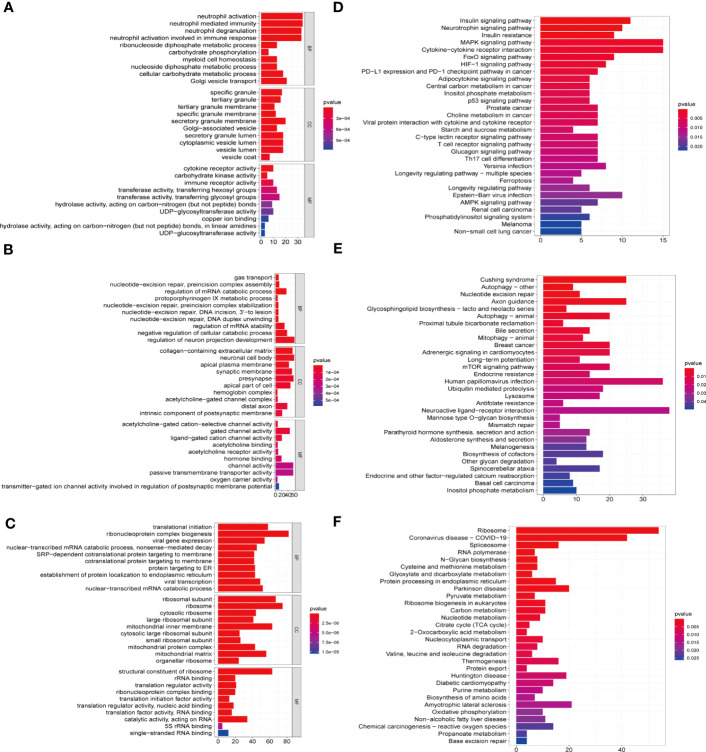
GO and KEGG enrichment analysis of eigengenes from the key module in C1-C3. GO functional enrichment analysis of the intersecting genes with the top 10, including molecular functions(MF), biological processes (BP) and cellular components (CC) terms and KEGG pathways. The horizontal axis shows the number of genes and the vertical shows the GO and KEGG terms. The color depth of the barplots represents the *p*-value. **(A, D)** GO and KEGG enrichment analysis of genes from MEblack module in cluster 1. **(B, E)** GO and KEGG enrichment analysis of genes from the MEblue module in cluster 2. **(C, F)** GO and KEGG enrichment analysis of genes from the MEyellow module in cluster 3.

To further explore the biological function of each module, we conducted GO and KEGG enrichment analyses (**Figures 6A–F**). GO enrichment analysis demonstrated that the biological functions of MEblack module (correlated with cluster 1) were mainly enriched in Neutrophil activation (**Figure 6A**); the MEblue module (correlated with cluster 2) was mainly related to cellular catabolic processes and mRNA stability (**Figure 6B**); and the MEyellow module (correlated with cluster 3) was mainly associated with Ribonucleoprotein complex biogenesis and carbohydrate metabolism pathways (**Figure 6C**). KEGG enrichment analysis showed that MEblack module (correlated with cluster 1) was mainly enriched in MAPK signaling pathway and cytokine-cytokine receptor interaction signaling pathway (**Figure 6D**); the MEblue module (correlated with cluster 2) was mainly related to Cushing syndrome and autophagy (**Figure 6E**); and the MEyellow module (correlated with cluster 3) was mainly associated with ribosome and Coronavirus disease-COVID-19 (**Figure 6F**).

### 3.5 Five hub RMGs showed diagnostic value for sepsis based on machine learning

**Figure 7:**
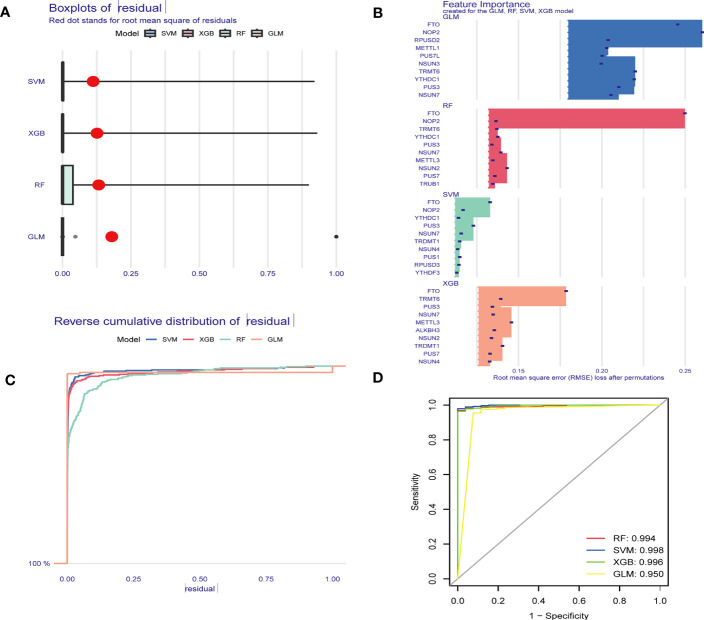
Construction and assessment of RF, GLM, SVM and XGB model. **(A)** Boxplots displaying the residuals of the sample, with the red dot indicating the root mean square of the residuals. **(B)** The feature importance of the variables in the RF, GLM, SVM and XGB model. **(C)** Cumulative residual distribution map of the sample. **(D)** ROC evaluation of the performance of the RF, GLM, SVM and XGB models. RF Random Forest, SVM Support Vector Machine, XGB eXtreme Gradient Boosting, GLM generalized linear models, ROC receiver operating characteristic.

**Figure 8:**
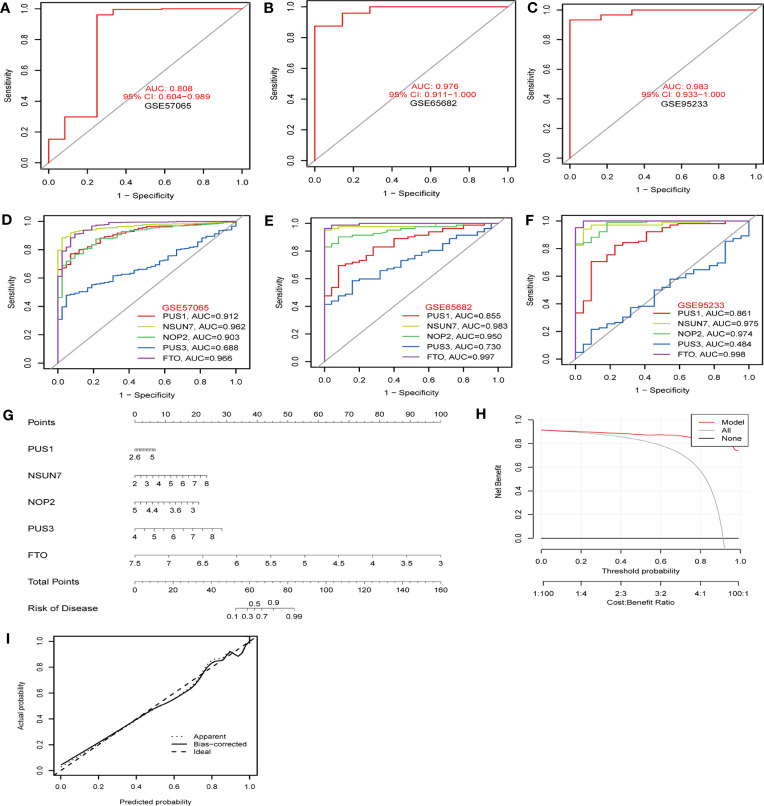
The diagnostic efficacy of hub RMGs. **(A–C)** The diagnostic efficacy of the SVM model in the discovery cohorts. **(A)** GSE57065 datasets. **(B)** GSE65682 datasets. **(C)** GSE95233 datasets. **(D–F)** ROC curves show the sepsis diagnostic efficacy of *NSUN7*, *FTO*, *NOP2*, *PUS1*, *PUS3* respectively. **(D)** GSE57065 datasets. **(E)** GSE65682 datasets. **(F)** GSE95233 datasets. **(G)** Nomogram to predict the occurrence of sepsis. **(H)** Decision curve analysis was applied to evaluate the clinical value of the nomogram model. The Y-axis represents the net benefit. The black line represents the hypothesis that no patients die. The X-axis represents the threshold probability, where the expected benefit of treatment equals the expected benefit of avoiding treatment. **(I)** Calibration curve indicates the predictive power of the nomogram model.

To identify hub RMGs with diagnostic value for sepsis, firstly, we refined the selection of RMGs by four algorithms, namely the RF, SVM, XGB and GLM, and used differentially expressed RMGs in **Figure 2A** to construct the four diagnostic models. We further evaluated the efficiency of the four models by usage of “DALEX” R package. As shown in **Figures 7A–C**, SVM model was the most suitable model for its least sample residual. In addition, the performance of the four models was evaluated by using ROC curves, SVM model displayed the highest AUC value (AUC=0.998) (**Figure 7D**). To further assess the accuracy and reliability of the SVM model, we conducted ROC analysis of three GEO databases. The AUC amount to 0.808 in GSE57065 (**Figure 8A**), 0.976 in GSE65682 (**Figure 8B**) and 0.983 in GSE95233 (**Figure 8C**). Therefore, the above results indicated that the SVM diagnostic model exhibited excellent predictive efficiency.

Subsequently, according to the feature importance of models in **Figure 6B**, the top 5 genes (*NSUN7*, *FTO*, *NOP2*, *PUS1*, and *PUS3*) selected by SVM were used for the analysis of diagnostic performance. AUC values of the ROC of these signature genes were 0.912 for *PUS1*, 0.962 for *NSUN7*, 0.903 for *NOP2*, 0.688 for *PUS3*, and 0.966 for *FTO* in GSE57065 (**Figure 8D**). In GSE65682, the AUC values of ROC were 0.855 for *PUS1*, 0.983 for *NSUN7*, 0.950 for *NOP2*, 0.730 for *PUS3*, and 0.997 for *FTO*, respectively (**Figure 8E**). Finally, the AUC values of ROC were 0.861 for *PUS1*, 0.975 for *NSUN7*, 0.974 for *NOP2*, 0.484 for *PUS3*, and 0.998 for *FTO* in GSE95233 (**Figure 8F**). **Table S9** reports the specificity, sensitivity and optimal cutoff point for discriminating between sepsis and healthy controls in GSE57065, GSE65682, and GSE95233, respectively. These results indicated that the screened signature genes particularly *NSUN7* and *FTO* exhibited remarkable diagnostic efficiency in sepsis.

To predict the occurrence of sepsis, we constructed a nomogram model for diagnosing sepsis based on the expression levels of five hub RMGs (*NSUN7*, *FTO*, *NOP2*, *PUS1* and *PUS3*) (**Figure 8G**). Decision curve analysis (DCA) revealed that the “nomogram model” curve was superior to the gray line, suggesting that the patients could benefit from the nomogram model at high-risk threshold from 0 to 1 and the clinical advantage of the nomogram model was higher (**Figure 8H**). We further assessed the predictive power of nomogram model *via* a calibration curve. The calibration curve showed no significant difference between the actual sepsis risk and the predicted risk, indicating that this model was accurate in predicting sepsis (**Figure 8I**). These findings suggested that the five hub genes are efficient for the diagnosis of sepsis.

### 3.6 Correlation between hub RMGs and immune cells in sepsis

**Figure 9:**
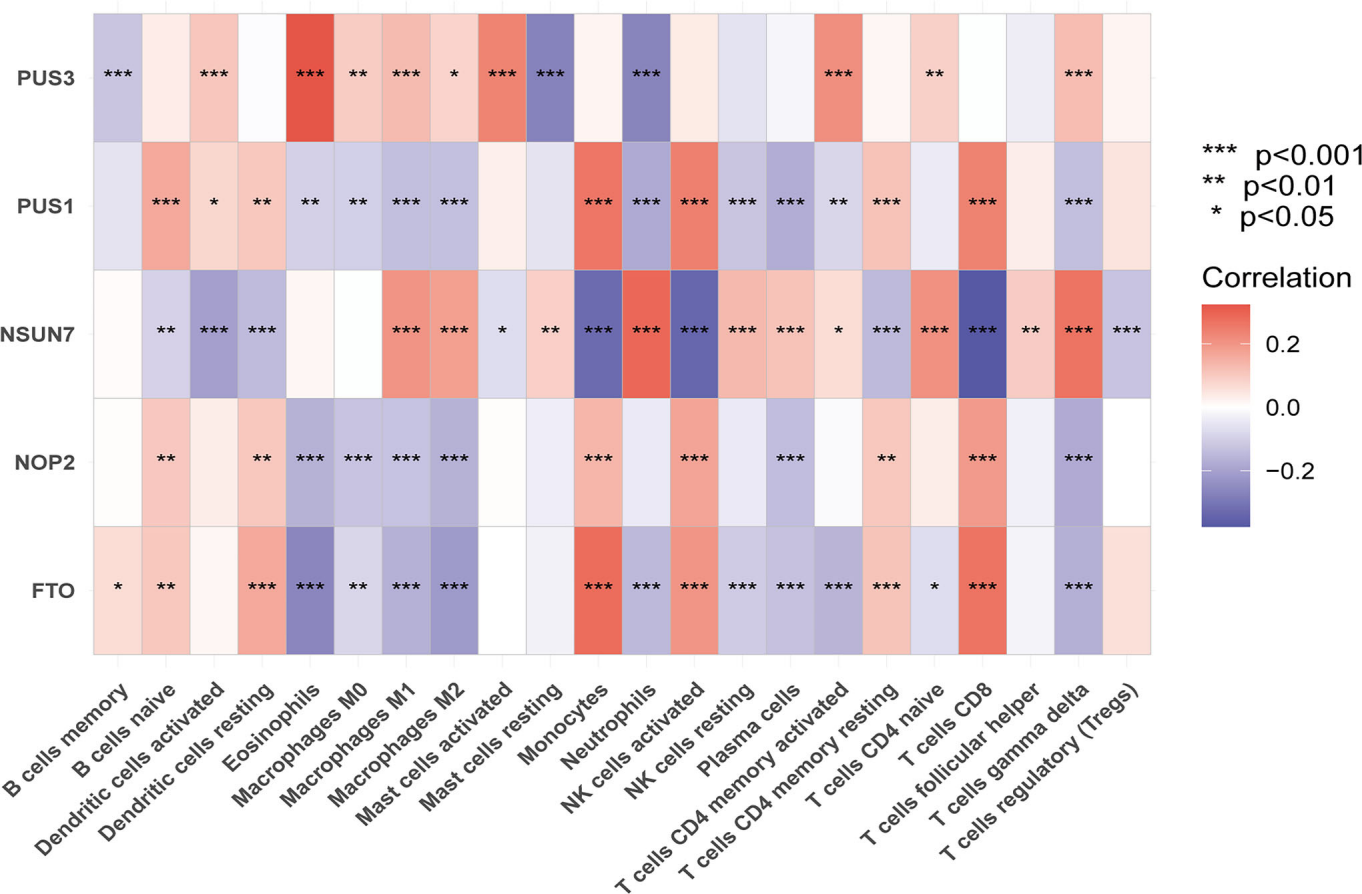
The correlation between hub RMGs and infiltrating immune cells in GEO datasets. *P < 0.01, **P < 0.001 and ***P < 0.0001.

We further investigated whether the hub RMGs were associated with immune cell infiltration in sepsis (**Figure 9**). We observed that *PUS3* was negatively correlated with resting mast cells and neutrophils. *PUS1* was negatively correlated with M1 macrophages, M2 macrophages, neutrophils, plasma cells and γδT cells, but positively correlated with monocytes, activated NK cells and CD8 T cells. Interestingly, similar to *PUS1*, *NOP2* and *FTO* were also positively correlated with monocytes, activated NK cells and CD8 T cells. Conversely, *NSUN7* displayed a negative correlation with monocytes, activated NK cells and CD8 T cells, but positive correlation with neutrophils, γδT cells and M1 macrophages. These results suggested that *PUS1*, *NOP2*, and *FTO* may act synergistically in sepsis, while *NSUN7* may counteract the function of aforementioned three genes.

Furthermore, by determining the expression of hub genes from single-cell transcriptome in Human Protein Atlas database (https://www.proteinatlas.org ), we found that *NOP2*, *FTO*, *PUS1*, and *PUS3* were all enriched in T cells, however, the *NSUN7* gene is not expressed in any immune cells in PBMCs from healthy subjects (**Figure S3**). In addition, to better understand the genes and microRNA associated with key RMGs, we constructed a gene co-expression analysis and mRNA-miRNAs networks (**Figure S4**, **Table S10**-**S11**), suggesting that *NOP2* and *PUS1* may be functionally synergistic with each other. Genes associated with hub RMGs may linked to the progression of sepsis, *NOP2* and *NAT10* were co-expressed, and previous research reported that *NAT10* improves the survival and ameliorates lung injury in septic mice by inhibiting neutrophil pyroptosis (32). We found miRNAs such as miR-21-3p (33) and miR-126-5p (34) were also crucial components in regulating the sepsis pathogenesis by interacting with key RMGs.

### 3.7 The validation of diagnostic value of the five hub RMGs in clinical settings

To validate the expression and diagnostic potential of the five hub RMGs in clinical samples, we prospectively recruited patients with sepsis and healthy controls. The general clinical characteristics of the patients was shown in **Table 1**. The cohort was comprised of 11 patients with septic shock, 15 sepsis patients without shock (sepsis), and 13 healthy volunteers. There were no significant differences in sex or age between the two (septic shock and sepsis) (*P* = 0.0965, *P* = 0.0740) or three groups (septic shock, sepsis and health) (*P* = 0.0501, *P* = 0.0772). Additionally, there were no significant differences in comorbidities or sites of infection between the two groups (septic shock and sepsis). Compared to the sepsis group, the patients in the shock groups exhibited higher Sequential Organ Failure Assessment (SOFA) and Acute Physiology and Chronic Health Evaluation (APACH) II scores (*P* = 0.0020, *P* = 0.0001), longer Mechanical ventilation time and higher mortality rate at day 7, 14 and 28 (*P* = 0.0177, *P* = 0.0020, *P* = 0.0038, *P* = 0.0008).

**Figure 10:**
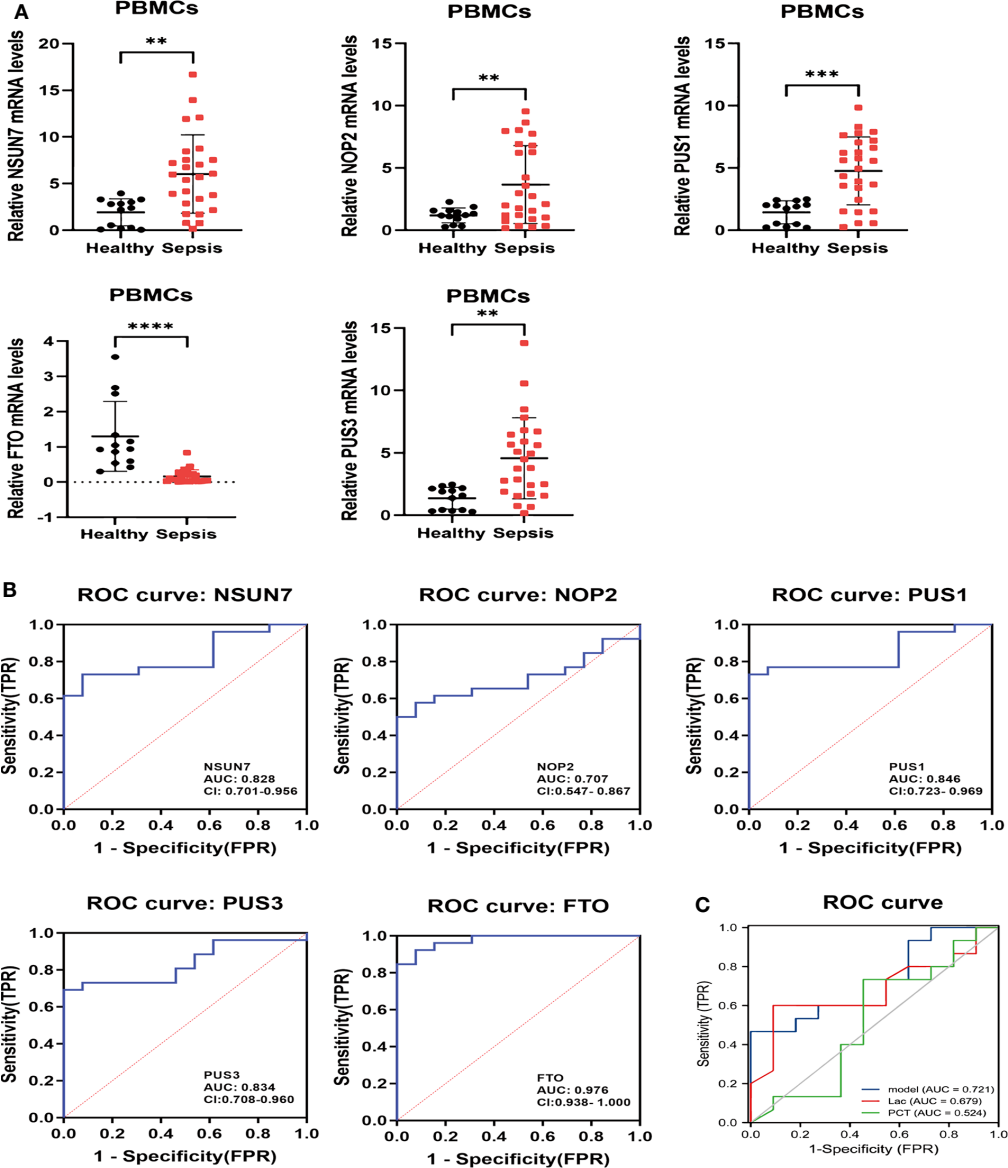
The relative expressions and ROC of hub RMGs in patients. **(A)** The concentrations of *NSUN7*, *NOP2*, *PUS1*, *PUS3* and *FTO* mRNA in PBMCs were measured by qRT-PCR from septic patients and healthy controls at day 1 after enrollment. **P < 0.01, ***P < 0.001, and ****P < 0.0001 vs. Healthy. **(B)** ROC curves of *NSUN7*, *NOP2*, *PUS1*, *PUS3* and *FTO* for diagnostic at admission in healthy and septic patients. **(C)** ROC curves illustrating the diagnostic performance of the integrated hub RMGs, Lac and PCT in sepsis and septic shock patients. Model the combined of *NSUN7*, *NOP2*, *PUS1*, *PUS3* and *FTO*. Lac Lactic acid. PCT procalcitonin.

Firstly, we determined the genes expression in PBMCs by qRT-PCR analysis, and found the expression of *NSUN7*, *NOP2*, *PUS1* and *PUS3* in septic patients (septic shock and sepsis) at day 1 were significantly higher than in healthy volunteers, and *FTO* expression was lower in septic patients (**Figure 10A**). However, the expression levels of the five genes displayed no significant difference in survivors and non-survivors in septic patients at day 28 (**Figure S5A**). In addition, their expression did not show any significant difference between septic shock and sepsis patients (**Figure S5B**). Notably, the expression of *NOP2* and *PUS1* was concurrently increased in patients with sepsis of group C3 in the integrated gene expression matrix (**Figure 2F**), which was consistent with the results observed in our clinic samples.

Then, we investigated the diagnostic value of hub RMGs in sepsis using ROC analysis. The AUC of *NSUN7*, *NOP2*, *PUS1*, *PUS3* and *FTO* at day 1 was 0.828, 0.707, 0.846, 0.834 and 0.976 respectively (**Figure 10B**). Notably, *FTO* exhibited the highest diagnostic value among these genes, with sensitivity of 0.846 and specificity of 1 (**Table S12**). Furthermore, we evaluated the combined diagnostic value of *NSUN7*, *NOP2*, *PUS1*, *PUS3* and *FTO* (the integrated RMGs model) among non-septic shock and septic shock patients. We compared the AUC of integrated RMGs model with Lactate (Lac) and procalcitonin (PCT) at day 1 and found that the AUC of the integrated RMGs model was not inferior to Lac and PCT (0.72 *vs*. 0.68, *P* = 0.78; 0.72 *vs*. 0.52, *P* = 0.24) (**Figure 10C**), with a sensitivity of 0.467 and specificity of 1 (**Table S13**).

## 4 Discussion

Sepsis is a complex and heterogenous clinical syndrome that requires extensive research to validate biomarkers, facilitate diagnosis and identify distinct molecular subtypes. By combination of bioinformatics analysis and clinical investigations, our study identified three distinct subtypes of sepsis based on the expression levels of the most significantly aberrant RMGs. Firstly, we identified genes that were characteristically expressed in each subgroup. In cluster 1, the genes *NSUN7* and *TRMT6* showed the highest levels of upregulation, and they were involved in the regulation of m5C and m1A RNA methylation modifications. In cluster 2, there was a significant increase in the expression of *NSUN3*, which plays a role in RNA m5C modification. Additionally, cluster 3 demonstrated a significant increase in the expression of *NSUN5* and *NSUN6*, both of which were associated with m5C modification. Next, the CIBERSORT algorithm was utilized to comprehensively assess the distribution of infiltrating immune cells in distinct subtypes. In cluster 1, there was a relatively increased frequency of neutrophils and γδT cells, while CD8 T cells, Tregs and activated NK cells were down-regulated. In contrast, cluster 2 and 3 exhibited opposing immune cell distribution patterns compared to cluster 1. In cluster 3, CD8 T cells and monocytes were observed to be relatively upregulated, while γδT cells and neutrophils showed down-regulation. Then, through GSVA, GO and KEGG analyses, we explored molecular pathways among the three clusters. In Cluster 1, we observed enhanced neutrophil and inflammatory signaling pathways activation, such as the Leukocyte transendothelial migration and MAPK signaling pathway. Cluster 2 was featured by the regulation of mRNA stability and amino acid metabolism. cluster 3 was primarily associated with ribonucleoprotein complex biogenesis and carbohydrate metabolism pathways. We also identified RMGs that may have diagnostic value for sepsis. To our knowledge, this is the first study that focused on investigating the classification and diagnostic value of RNA methylation (including m5C, m6Am, m1A, m7G and Ψ) in sepsis by transcriptome-wide mapping.

To start with, we observed the expressions of various RNA methylation modifying enzymes in PBMCs from sepsis patients and found that RNA m5C modification may be involved in the pathology of sepsis. An active methyl-group from the donor, usually S-adenosyl-methionine (SAM), is added to the carbon-5 position of the cytosine base in RNA to form the m5C modification (35), which exerts biological functions including regulation of inflammatory response. In hyperhomocysteinemia, *NSUN2* upregulates IL-17A expression by mediating IL-17A mRNA m5C modification in T lymphocytes to induce chronic inflammation (36). In SLE, the m5C level and *NSUN2* expression are decreased in CD4^+^ T cells, and hypermethylated m5C is related to inflammatory pathways (37). Till now, the role of RNA m5C modification in sepsis has not been reported. Our study indicates that RNA m5C may be involved in sepsis and the underlying mechanism of this modification in the pathogenesis of sepsis still need further investigation.

We also discovered that immune-activated status and metabolic mechanisms (amino acid and carbohydrate metabolism) may be regulated by RNA methylation in sepsis. Our results showed that *NSUN7* (m5C gene) and *TRMT6* (m1A gene) were increased in cluster 1, accompanied by neutrophil activation and upregulation of the MAPK signaling pathway. Neutrophil activation in sepsis is a complex process, and dysregulation of MAPK signaling can contribute to excessive neutrophil activation (38). In cluster 2, the physiological function analysis demonstrated that *NSUN3* may be involved in amino acid metabolism in sepsis. Untargeted metabolomics analysis revealed widespread dysregulation of amino acid metabolism in patients with sepsis, which regulates inflammation and immunity (39). In addition, *NSUN3* and m5C RNA methylation modification has been established to regulate metabolism in metastasis (40), which is in consistent with our findings in sepsis. Our analysis further revealed that the cluster 3 exhibited elevated levels of *NSUN5* and *NSUN6*, which primarily involved in carbohydrate metabolism. Recently, an increasing number of studies have indicated that glycolysis plays a crucial role in regulating both innate and adaptive inflammatory responses. Inhibition of glycolysis can reduce the levels of inflammatory cytokines and lactate expression in the myocardium of septic mice and improve cardiac function (41, 42). Furthermore, RNA methylation has been demonstrated to regulate glycolysis in tumors and immune diseases (43, 44). In summary, our study provides insights into the heterogeneity of sepsis, which is largely associated with m5C RNA methylation and involves immune and metabolic mechanisms, the underlying molecular mechanism of m5C RNA modification in regulation of sepsis needs further investigation.

Finally, by using machine learning, we identified five hub RMGs (*NSUN7*, *FTO*, *NOP2*, *PUS1*, and *PUS3*), which showed efficient diagnostic value of sepsis. We also evaluated the diagnostic value by combination of the five genes, and illustrated that the combined diagnostic value was not inferior to the value of classical biomarkers as Lac or PCT for septic shock. *NSUN7*, a member of the NSUN methyltransferase family, reduces protein activity and motility of sperms and is associated with male infertility (45). A recent study has reported that *NSUN7* is up-regulated in neonatal sepsis, combined with bioinformatic analyses, *NSUN7* is closely related to immune and inflammatory responses, implying its potential as a biomarker for the pathogenesis of neonatal sepsis (46). Consistently, we verified *NSNU7* was highly expressed in adult septic patients compared to healthy controls. Meanwhile, our results found *NSUN7* displayed a negative correlation with monocytes, activated NK cells and CD8 T cells, but a positive correlation with neutrophils, γδT cells and M1 macrophages, suggesting that *NSUN7* may be related to the excessive inflammatory response in sepsis. *FTO* belongs to the AlkB protein family, and its expression is closely related to weight gain and obesity (47). It is well established that the expression of *FTO* is decreased in septic mice (48), and our results further found a low expression in septic patients. A recent investigation demonstrated that knockdown of *FTO* inhibited inflammatory factor secretion, improved organ damage and survival in septic mice (49), suggesting a correlation between *FTO* and the inflammatory response process of sepsis, implying its potential as a diagnostic or prognostic marker for sepsis. The *PUS3* is a type of enzyme that catalyzes the formation of Ψ38 in the anticodon loop of certain tRNAs (50). Mutations in the *PUS3* gene have been associated with intellectual disability (51). However, little is known about its expression and function in relation to infectious or inflammatory diseases. *PUS1* is responsible for the isomerization of uridine to pseudouridine in RNA molecules. Mutations in the *PUS1* gene have been linked to mitochondrial myopathy, encephalopathy, lactic acidosis, and stroke-like episodes (MELAS) (52). In our study, the expression of *PUS1* in the clinical validation and bioinformatics analysis results was not consistent, potentially due to patient heterogeneity and sample size. *NSUN1* is an RNA-binding protein that belongs to the NOP2/SUN (NSUN) RNA-methyltransferase family. Previous research suggests that *NOP2* is expressed at higher levels in human malignant tumor cells and is considered as a prognostic marker for cancer aggressiveness (53). Our clinical validation results are consistent with a previous study reporting a high *NOP2* expression in septic patients (54), and its role and mechanism in sepsis remain to be further explored experimentally. Overall, *NSUN7*, *NOP2*, *PUS1*, *PUS3*, and *FTO* were identified as important diagnostic markers for sepsis.

The present study provided possible regulatory relationships between RNA methylation regulators and sepsis. There are still some limitations to our study. First, our clinical study only included patients from a single center with a relatively small sample size. Further multi-center clinical studies with larger sample sizes are necessary to confirm the expression levels, diagnostic value for clinical use. Second, the specificity of the hub genes for diagnosing sepsis is high, but the sensitivity is relatively low. Future studies are needed by using enrolling non-septic patients with infection as control to assess the sensitivity and specificity of the diagnostic markers for sepsis. Third, *in vitro* and *in vivo* experiments are needed to explore the molecular mechanisms and identify the exact roles of the hub genes in sepsis.

## Data availability statement

Publicly available databases were analyzed in this study. This data can be found here: https://www.ncbi.nlm.nih.gov/geo/, GSE57065, GSE65682, and GSE95233.

## Ethics statement

This study was approved by the Research Ethics Board of East Hospital, Tongji University (Shanghai, China). Written informed consent was obtained from all recruited patients or their authorized family members.

## Author contributions

QZ designed the research, conducted experiments and wrote the manuscript. XB collected baseline information of clinical patients. QZ and JLJ performed data analysis. MC, CW and JJ contributed to the writing of the manuscript. All authors contributed to the article and approved the submitted version.

## Glossary

**Table:**

RMGs	RNA methylation genes
DEGs	Differentially expressed genes
GSVA	Gene Set Variation Analysis
GO	Gene ontology
KEGG	Kyoto Encyclopedia of Genes and Genomes
PPI	Protein–protein interaction
BP	Biological process
CC	Cellular component
MF	Molecular function
RF	Random Forest
SVM	Support Vector Machine
XGB	eXtreme Gradient Boosting
GLM	generalized linear models
ROC	receiver operating characteristics
AUC	area under the ROC curve
PBMCs	peripheral blood mononuclear cells
PBMCs	peripheral blood mononuclear cells
WGCNA	weighted correlation network analysis
m1A	N1-methyladenosine
m5C	5-methylcytosine
m6A	N6-methyladenosine
m6Am	2’-O-dimethyladenosine
m7G	N7-methylguanosine
Ψ	Pseudouridine
NSUN	Nol1/Nop2/Sun
FTO	fat mass and obesity-associated protein
PUS1	pseudouridine synthase 1 gene
PUS3	pseudouridine synthase 3 gene
NSUN7	NOP2/Sun RNA methyltransferase 7
NOP2	nucleolar protein P120
