# Supplementary material for: Identification and validation of key biomarkers based on RNA methylation genes in sepsis
Source: Front Immunol. 2023 Aug 28;14:1231898. doi: 10.3389/fimmu.2023.1231898 (PMC10493392; doi:10.3389/fimmu.2023.1231898)
Supplement: Supplementary file 1 [file DataSheet_1.zip › Supplementary Material Presentation/Supplementary Figures/Supplementary_Material Figures.docx]

Supplementary Material

Identification and validation of key biomarkers based on RNA Methylation genes in sepsis

Qianqian Zhang^1,2^, Xiaowei Bao^1,2^, Mintian Cui^3^, Chunxue Wang^1,2^, Jinlu Ji^1,2^, Jiongjie Jing^3^, Xiaohui Zhou^4^, Kun Chen^3,5*^, Lunxian Tang^1,2*^

*** Correspondence:** Corresponding Author:

Kun Chen, Email: [Chenk@tongji.edu.cn;](mailto:Chenk@tongji.edu.cn;) Lunxian Tang, Email: [456tlx@163.com](mailto:456tlx@163.com)

# Supplementary Figures

#
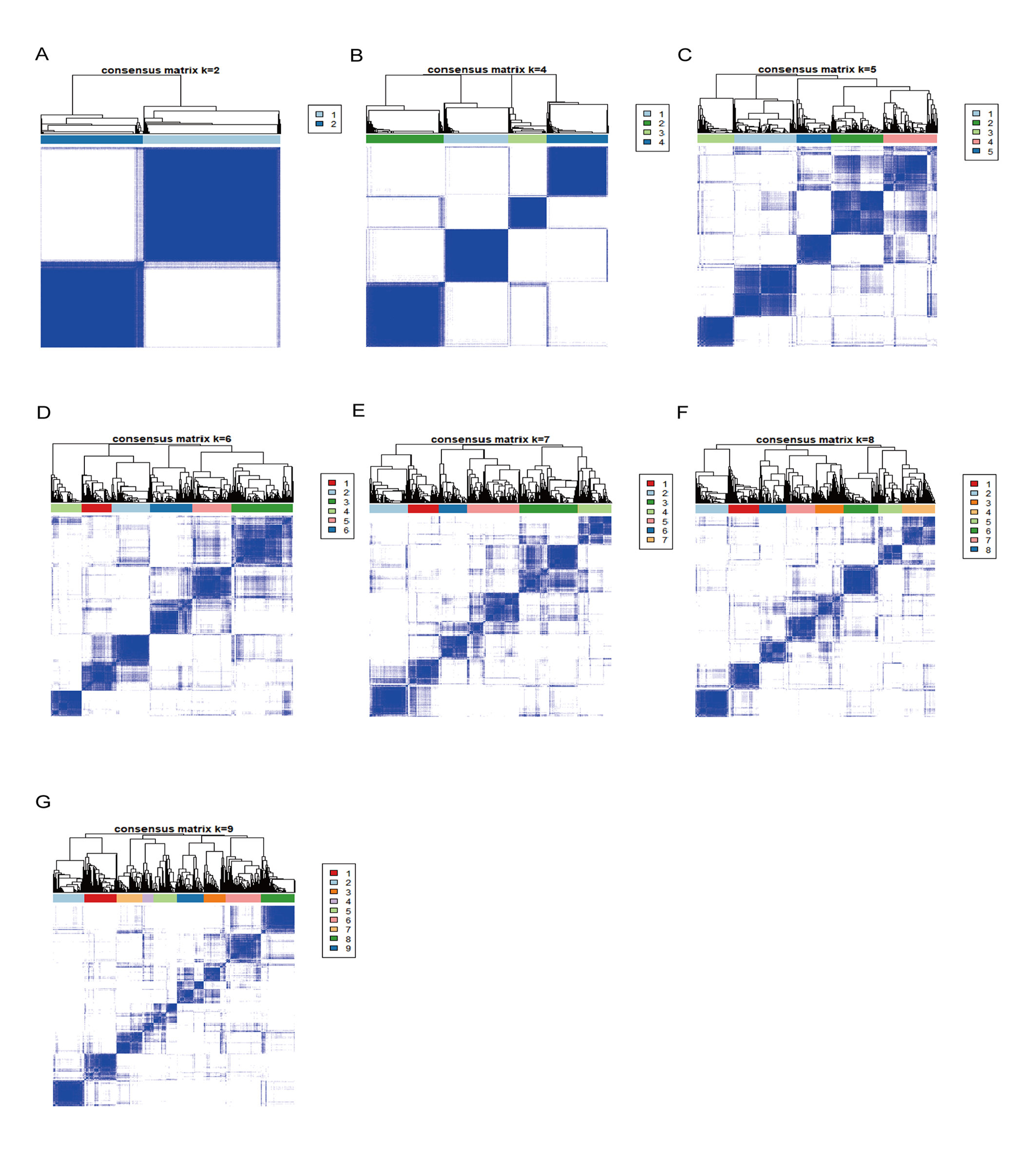


**Supplementary Figure 1** Unsupervised consensus clustering analysis for sepsis samples based on RMGs expression profiles. **(A-H)** Heatmaps of the matrix of co-occurrence proportions for k=2, 4-9.


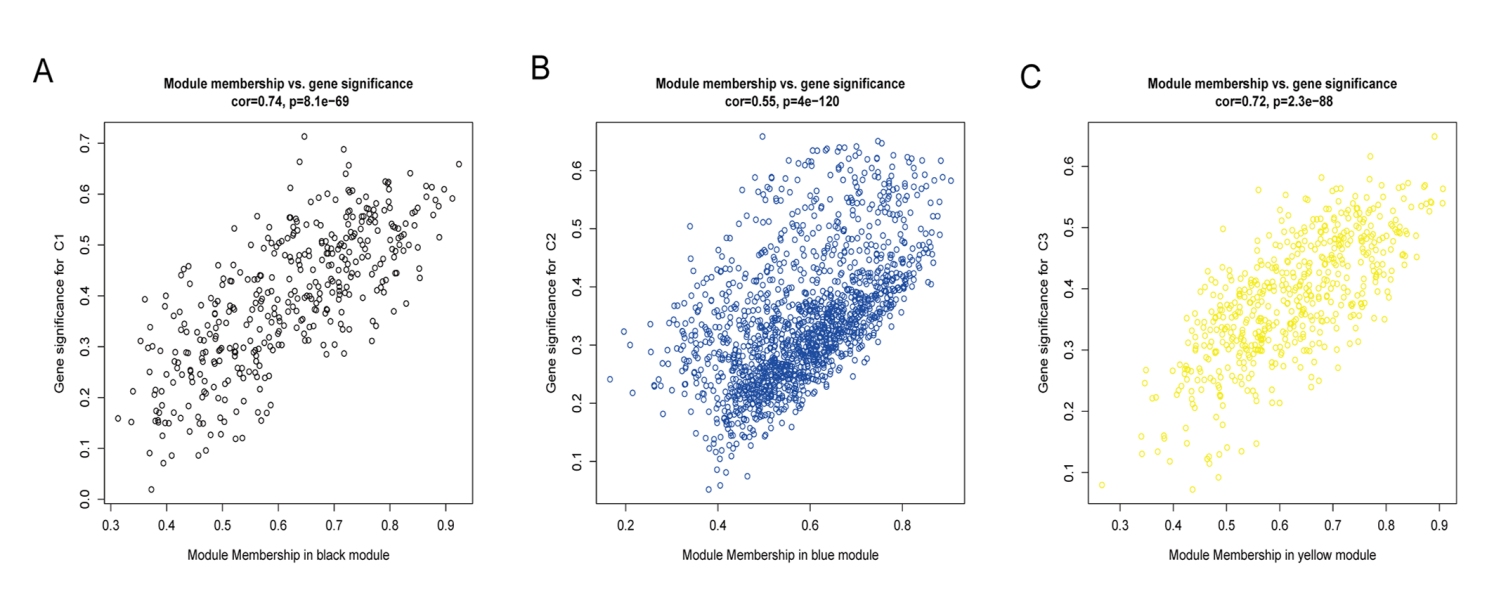


**Supplementary Figure 2** Correlation between module membership (X-axis) and gene signifcance. **(A)** Correlation between module membership (X-axis) and gene signifcance (Y-axis) of genes from the black module. **(B)** Correlation between module membership (X-axis) and gene signifcance (Y-axis) of genes from the blue module. **(C)** Correlation between module membership (X-axis) and gene signifcance (Y-axis) of genes from the yellow module.


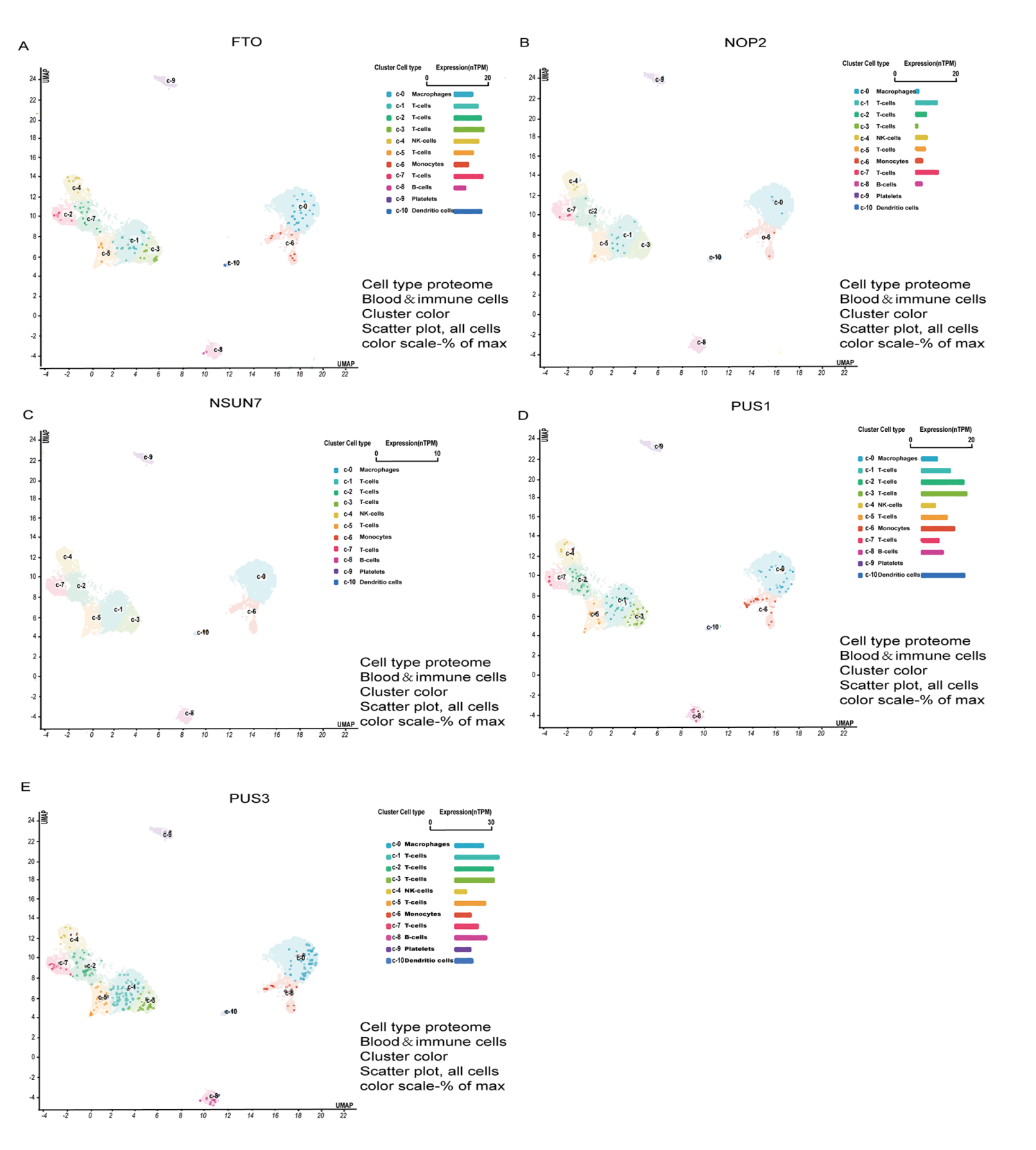


**Supplementary Figure 3** Analysis of FTO, NOP2, NSUN7, PUS1 and PUS3 expression in single cell types. The expression of five hub RMGs in single cell types was analyzed using the HPA single-cell sequencing online site. Interactive UMAP plots and summarizing bar plots display the expression of each gene in single cell types of healthy human peripheral blood mononuclear cells.


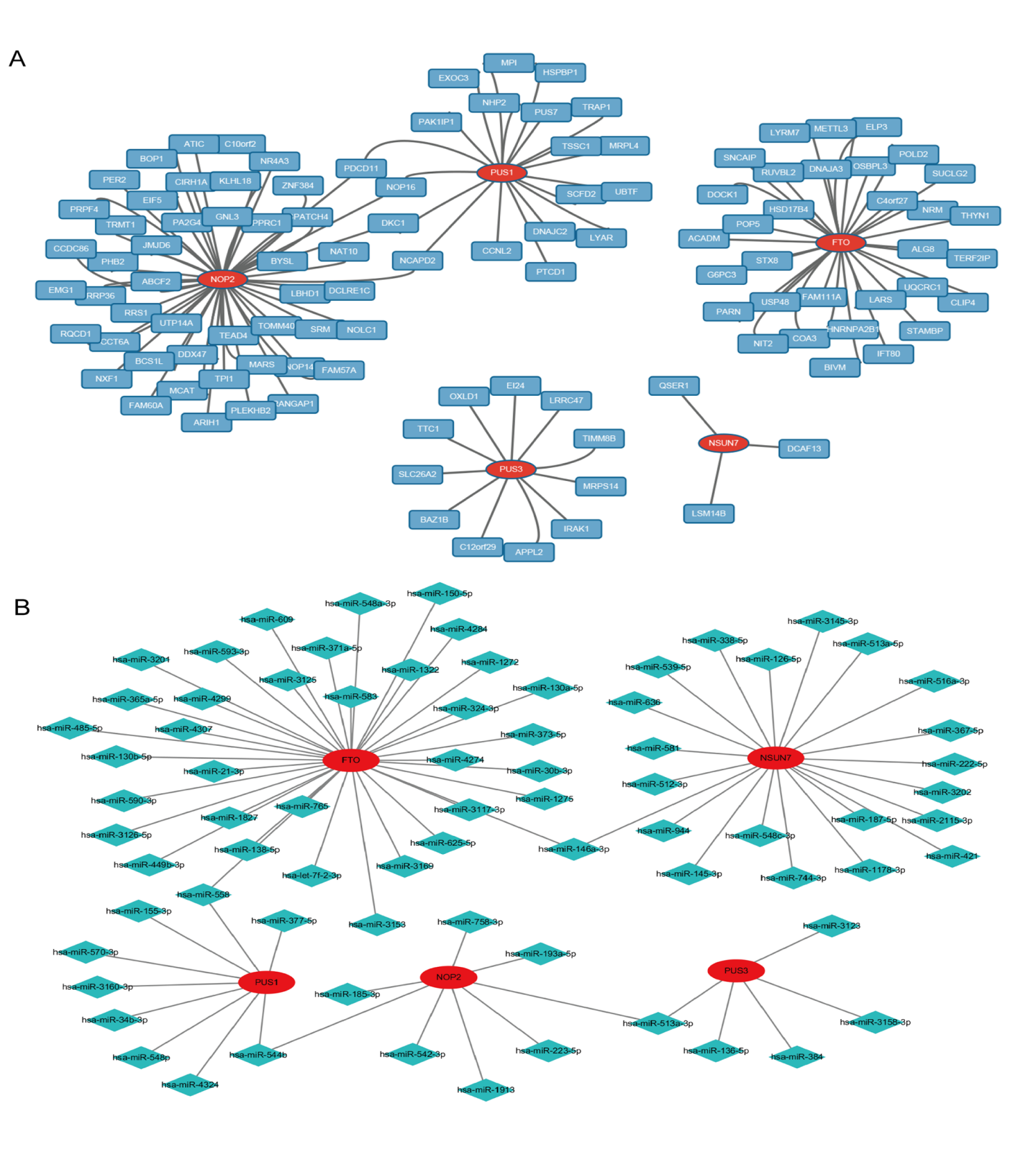


**Supplementary Figure 4** Co-expressed genes and miRNA-mRNA network construction. **(A)** The co-expression network of of hub genes in COEXPEDIA. Blue nodes indicate co-expressed gene, red nodes represent hub RMGs related to sepsis. **(B)** The miRNA-mRNA network of hub RMGs related to sepsis, with green nodes representing miRNAs and red nodes representing key genes related to sepsis.

**
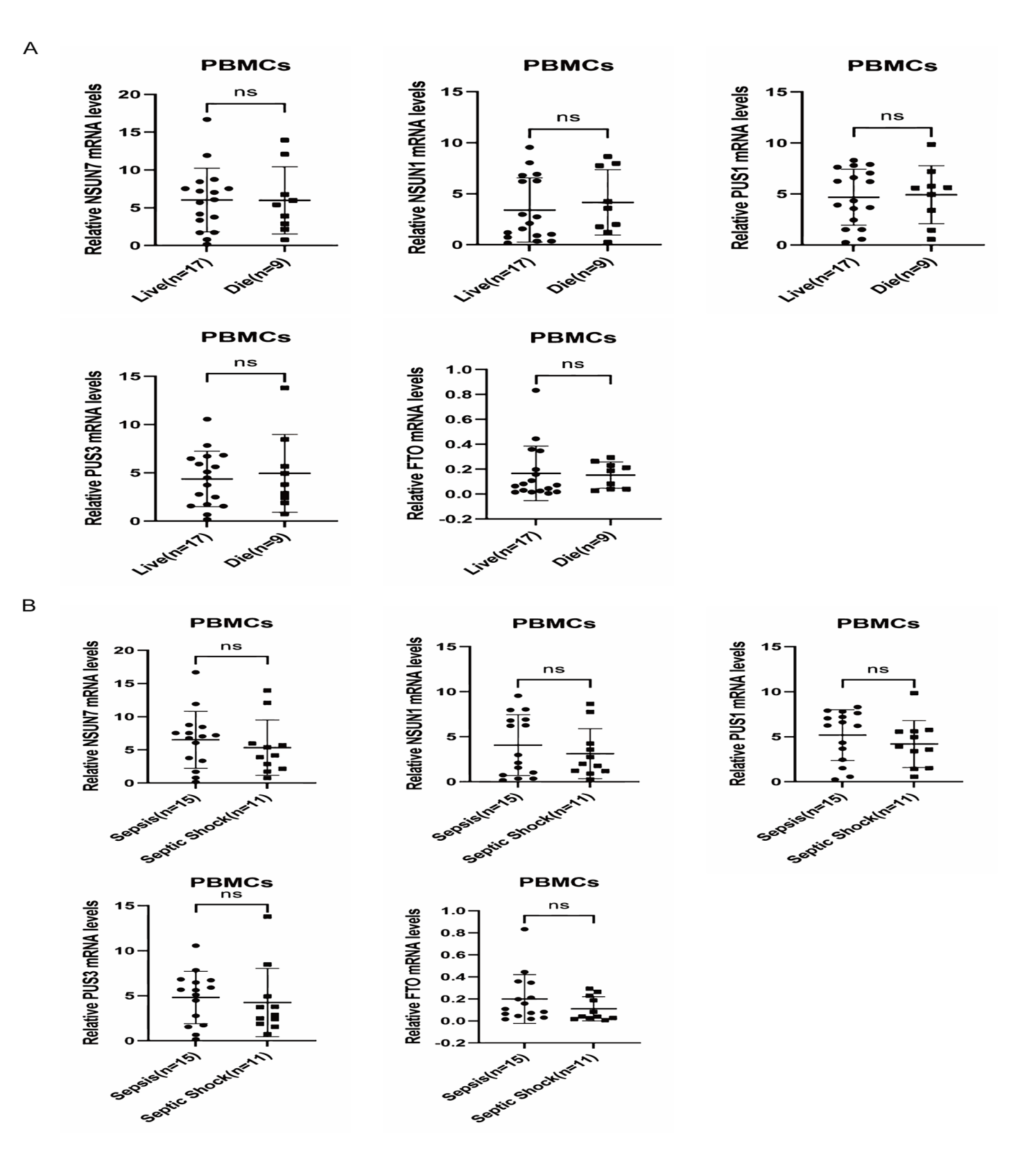
**

**Supplementary Figure 5** The relative expressions of diagnostic genes mRNA in patients. **(A)** The expression of PBMCs NSUN7, NOP2, PUS1, PUS3 and FTO from septic shock and non-septic shock patients at day 1. **(B)** The level of NSUN7, NOP2, PUS1, PUS3 and FTO between survivors and non-survivors in patients with sepsis at 28 days. Ns not signifcant.
