# Supplementary figures and images for: Identification and validation of key biomarkers based on RNA methylation genes in sepsis
Source: Front Immunol. 2023 Aug 28;14:1231898. doi: 10.3389/fimmu.2023.1231898 (PMC10493392; doi:10.3389/fimmu.2023.1231898)

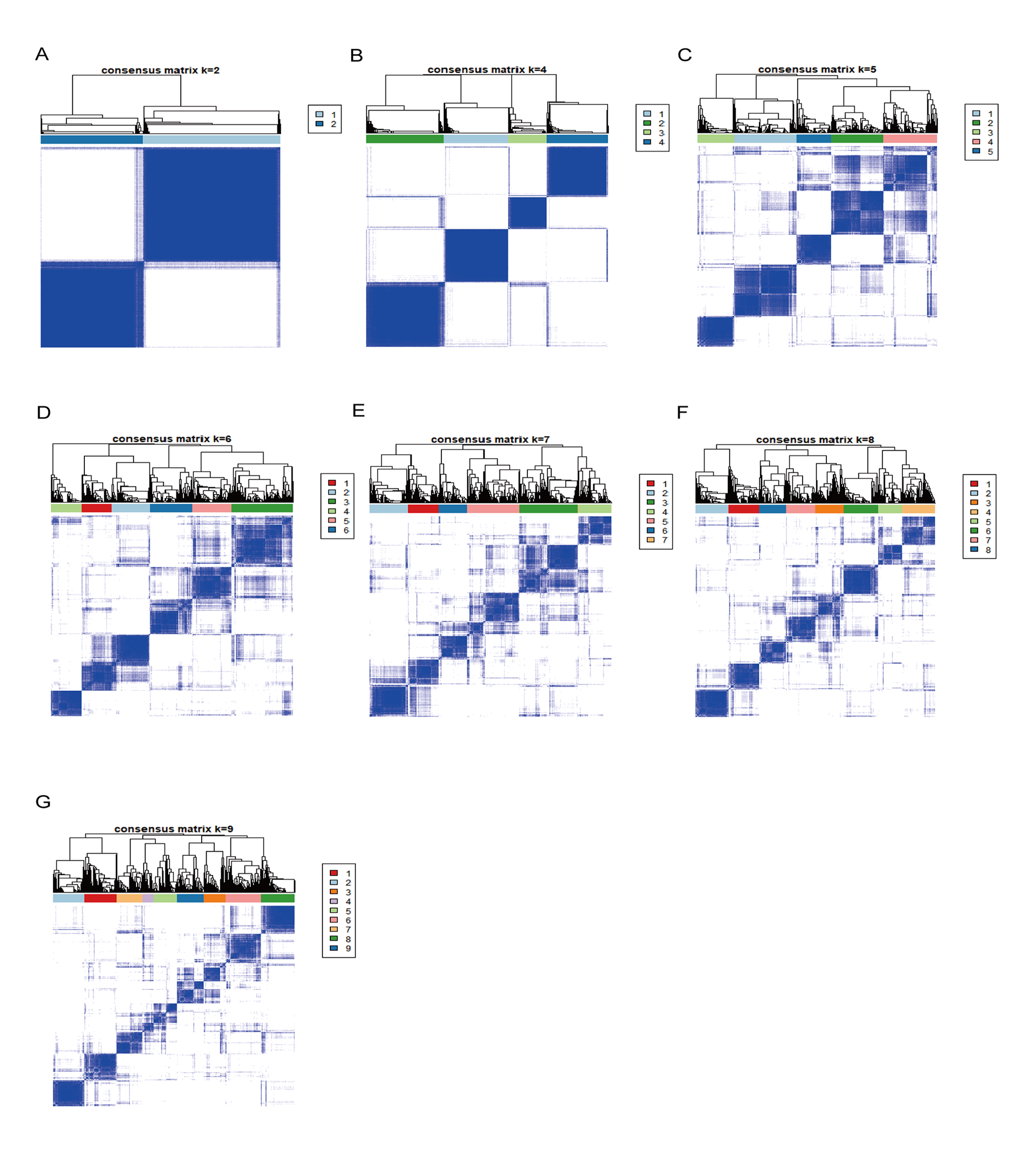

Supplement: Supplementary file 1 [file DataSheet_1.zip › Supplementary Material Presentation/Supplementary Figures/Supplementary Figure 1.tif]

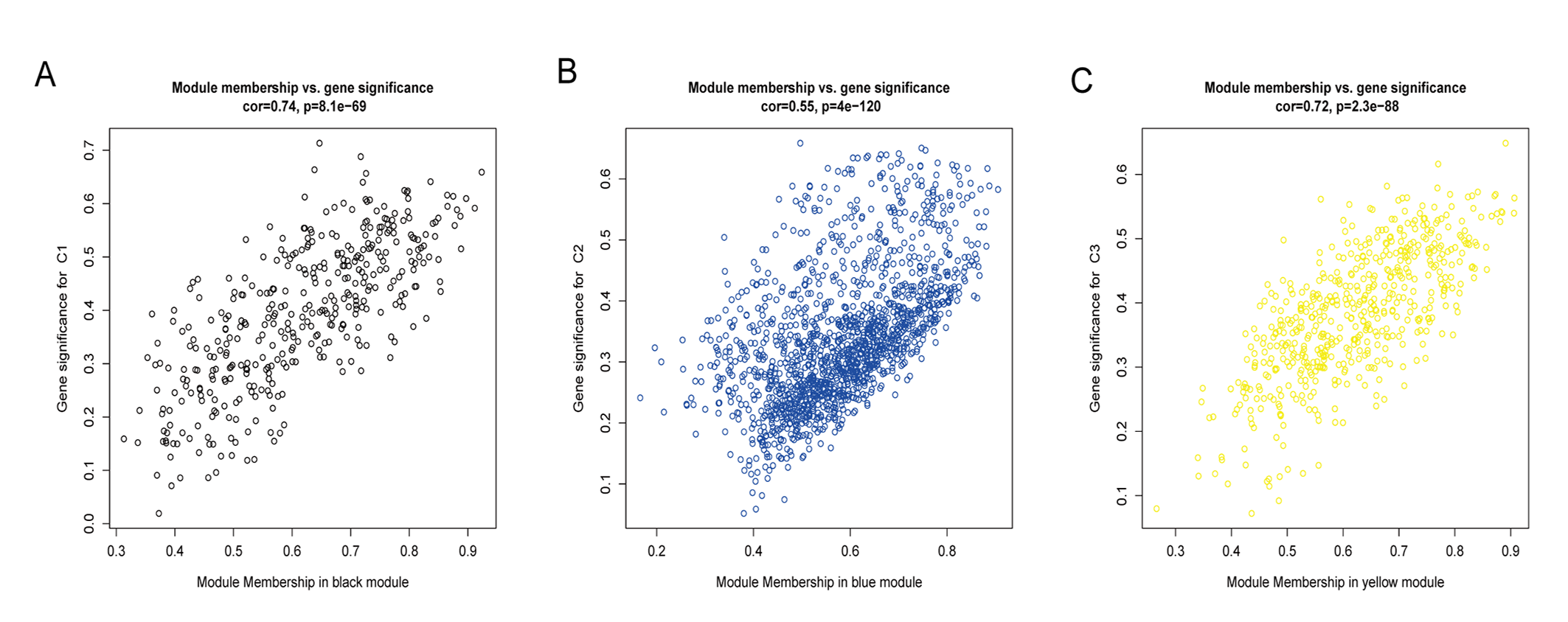

Supplement: Supplementary file 1 [file DataSheet_1.zip › Supplementary Material Presentation/Supplementary Figures/Supplementary Figure 2.tif]

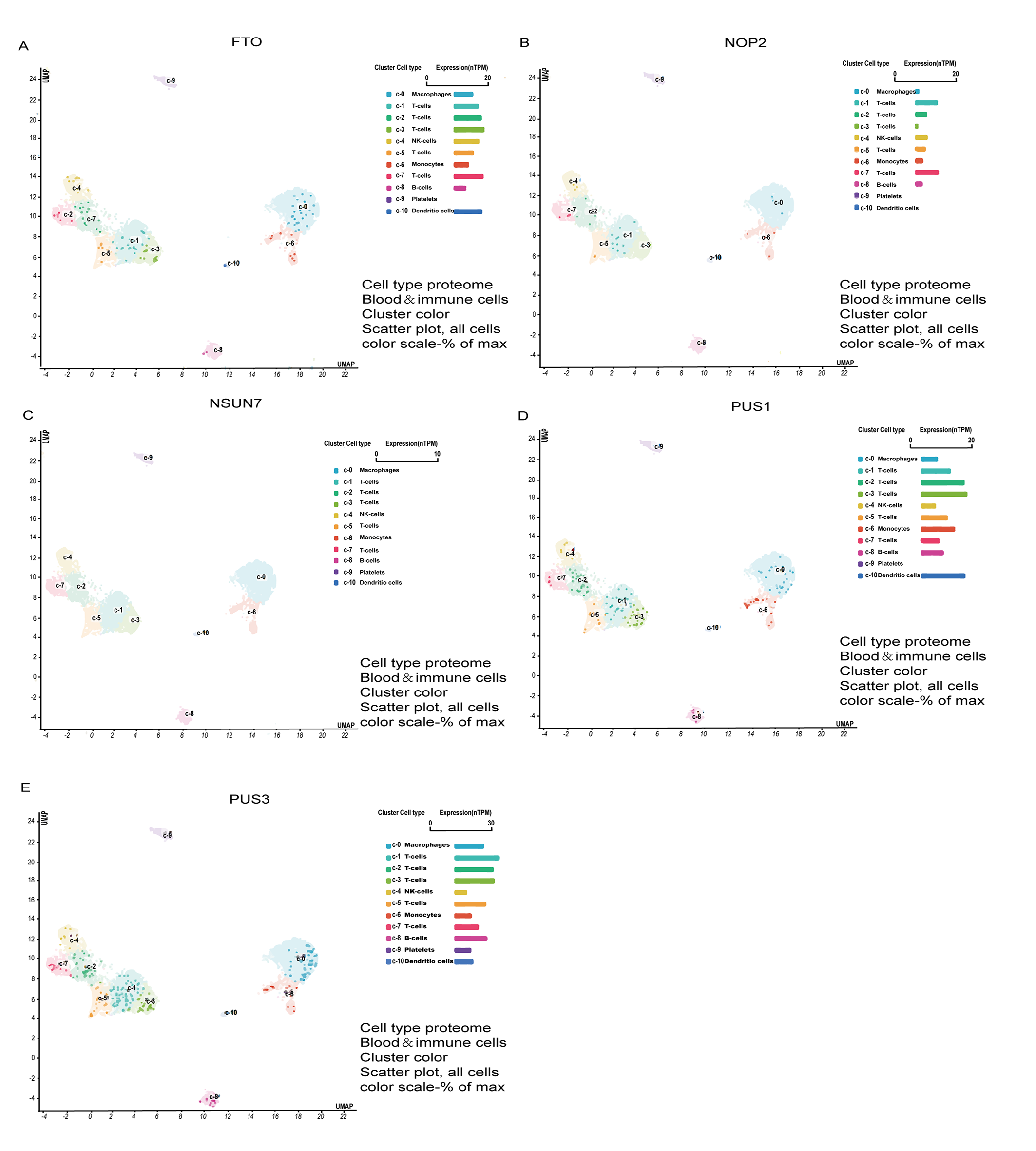

Supplement: Supplementary file 1 [file DataSheet_1.zip › Supplementary Material Presentation/Supplementary Figures/Supplementary Figure 3.tif]

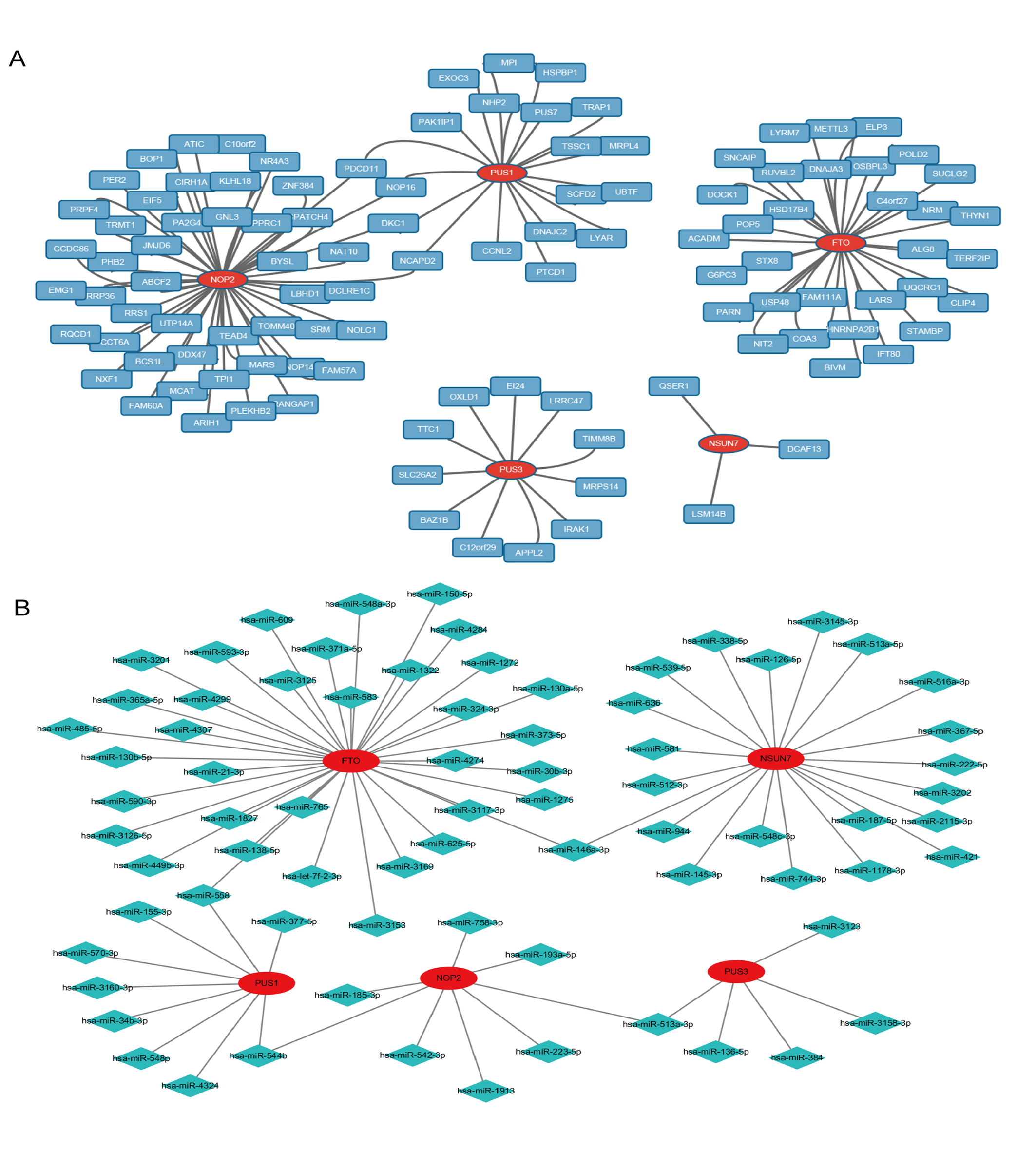

Supplement: Supplementary file 1 [file DataSheet_1.zip › Supplementary Material Presentation/Supplementary Figures/Supplementary Figure 4.tif]

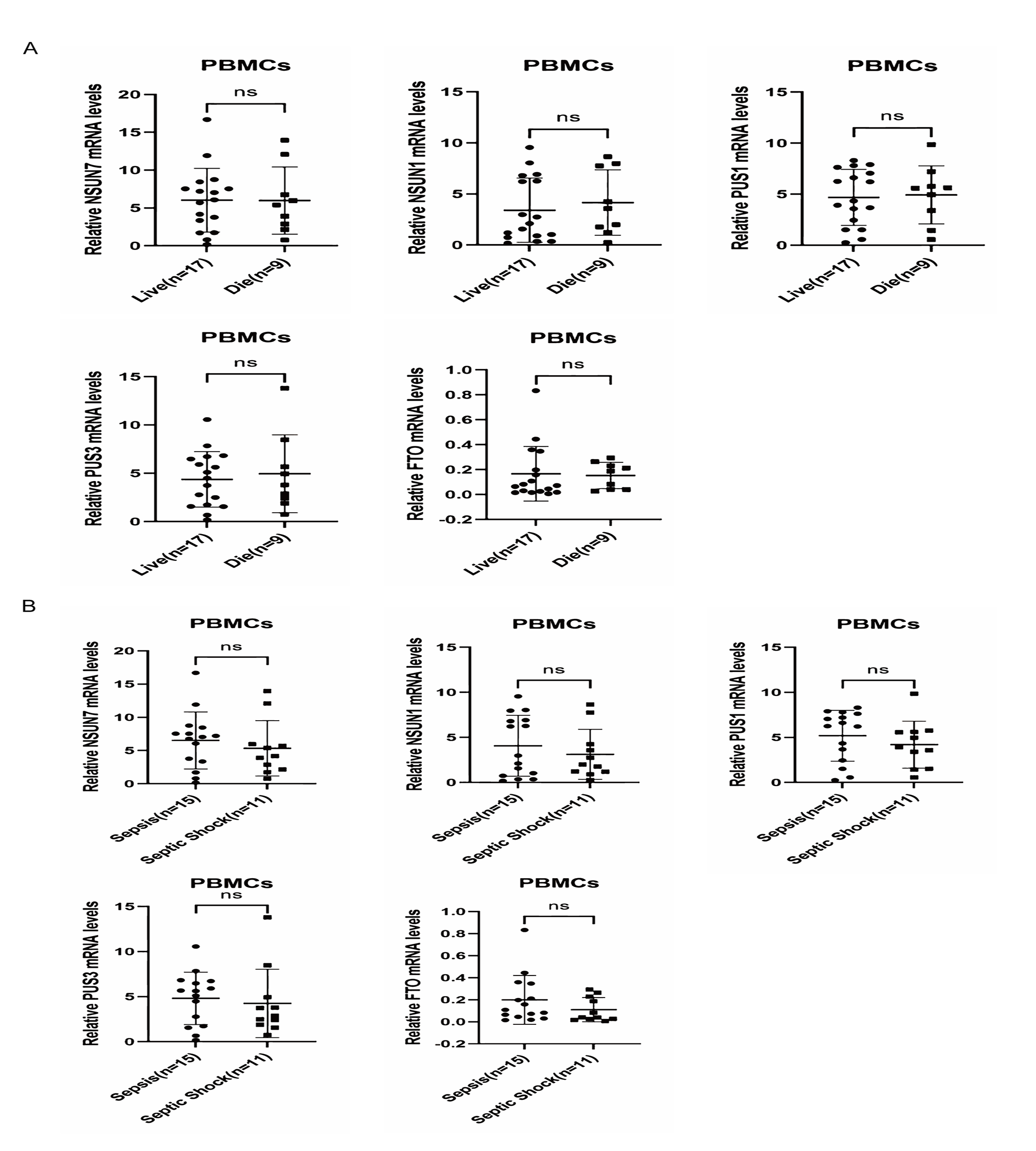

Supplement: Supplementary file 1 [file DataSheet_1.zip › Supplementary Material Presentation/Supplementary Figures/Supplementary Figure 5.tif]
